# SENP1-SIRT3 axis mediates glycolytic reprogramming to suppress inflammation during *Listeria monocytogenes* infection

**DOI:** 10.1128/mbio.02524-24

**Published:** 2025-03-12

**Authors:** Yan Xiong, Yongliang Du, Feng Lin, Beibei Fu, Dong Guo, Zhou Sha, Rong Tian, Rui Yao, Lulu Wang, Zixuan Cong, Bohao Li, Xiaoyuan Lin, Haibo Wu

**Affiliations:** 1 School of Life Sciences, Chongqing University 47913 , Chongqing, China; 2 Department of Pathology, Chongqing Hygeia Hospital, Chongqing, China; 3 Department of Clinical Microbiology and Immunology, College of Pharmacy and Medical Laboratory, Army Medical University (Third Military Medical University), Chongqing, China; The University of North Carolina at Chapel Hill School of Medicine, Chapel Hill, North Carolina, USA

**Keywords:** SENP1-SIRT3 axis, SUMO, glycolysis, PKM2, *Listeria monocytogenes*

## Abstract

*Listeria monocytogenes,* a foodborne pathogen, has the ability to invade intestinal mucosal cells, undergo intracellular proliferation, activate host immune responses, and induce diseases such as colitis. We have demonstrated that sentrin-specific protease 1 (SENP1) functions as a protective gene in the host, suppressing the inflammatory response triggered by *Listeria monocytogenes*. The host’s SENP1-SIRT3 axis plays a critical role in regulating inflammation during *Listeria monocytogenes* infection. Our findings reveal that overexpression of SENP1, particularly under *Listeria monocytogenes* infection conditions (MOI = 20), effectively suppresses inflammation through modulation of glycolysis. Mechanistically, during *Listeria monocytogenes* infection, SENP1 accumulates in the mitochondria, facilitating the de-SUMOylation and activation of sirtuin 3 (SIRT3). Activated SIRT3 then regulates the deacetylation of pyruvate kinase M2 (PKM2), leading to a decrease in glycolytic intermediates, downregulation of glycolysis-related gene expression, and suppression of inflammation. Taken together, our study provides a deeper understanding of the mechanistic role of the SENP1-SIRT3 axis in the regulation of inflammation, offering novel insights, and strategies for the treatment and prevention of inflammatory diseases.

**IMPORTANCE:**

Sentrin-specific protease 1 (SENP1)-sirtuin 3 (SIRT3) has never been reported in the regulation of bacteria-induced inflammation. Our study demonstrated that SENP1 acted as a protective factor against *Listeria*-induced inflammation by promoting SIRT3 activation and subsequent metabolic reprogramming. The SENP1-SIRT3 axis served not only as an essential signaling pathway for regulating mitochondrial metabolic responses to metabolic stress but also responds to bacterial invasion and plays a protective role in the organism. Our findings provide a basis for further research into targeting the SENP1-SIRT3 signaling pathway for the treatment of bacterial infections.

## INTRODUCTION

*Listeria monocytogenes*, a Gram-positive intracellular bacterium, is a foodborne pathogen capable of infecting humans and other animal species. It leads to gastroenteritis that might be complicated by bacteremia and central nervous system infection, which is associated with up to 30% mortality (1). *L*. *monocytogenes* triggers inflammation by invading host cells, evading immune detection, and releasing specific molecules recognized as foreign by the immune system. These trigger inflammatory responses, including the release of cytokines such as IL-1β, IL-18, and TNF-α, prompting immune cells to clear the infection (2). Immune cells regulate inflammation through energy metabolism, thereby maintaining tissue homeostasis. For example, regulatory T cells and M2 macrophages primarily acquire energy through fatty acid oxidation, particularly via the tricarboxylic acid cycle (TCA cycle). This process helps to suppress excessive inflammation and promotes tissue repair and immune tolerance (3, 4). Hence, our study focuses on elucidating the mechanism through which *L. monocytogenes* regulates inflammation via glycolysis.

Sentrin-specific protease 1 (SENP1) is a human protease of 643 amino acids with a weight of 73 kDa (5). SENP1 catalyzes the maturation of SUMO proteins (small ubiquitin-related modifiers), leading to the hydrolysis of the peptide bond in a conserved sequence Gly-Gly-|-Ala-Thr-Tyr at the C-terminal. This process results in the conjugation of SUMO to other proteins, a phenomenon known as sumoylation (6). Vertebrates possess three families of SUMO proteins: SUMO-1, SUMO-2, and SUMO-3. SENP1 is capable of catalyzing any of these three. The conjugation of SUMO to other proteins is analogous to ubiquitination; however, these modifications lead to different results depending on the type of protein being modified (7). Sirtuin 3 (SIRT3), a mitochondrial NAD-dependent deacetylase, is regulated by SUMOylation in mitochondria. SUMOylation suppresses SIRT3’s catalytic activity, while SENP1, a SUMO-specific protease, de-SUMOylates and activates SIRT3 (8). Some studies have indicated that the SUMO protease SENP1 promotes T cell memory development by desumoylating SIRT3. The SENP1-SIRT3 axis enhances SIRT3 deacetylase activity, resulting in increased oxidative phosphorylation and mitochondrial fusion. Mechanistically, SENP1 activates mitochondrial SIRT3 in T cells, reducing the acetylation of mitochondrial metalloprotease YME1L1 (9). Therefore, we hypothesize that SENP1 plays a crucial role in the regulation of glycolysis.

Pyruvate kinase M2 (PKM2), as a crucial enzyme in the glycolytic pathway, exhibits various biological effects and different configurations (four isoforms: L, R, M1, and M2) (10, 11). Particularly, the acetylation modification of PKM2 significantly influences its activity, protein interactions, and intracellular localization. The transition of PKM2 configuration from tetramer to dimer not only impacts its role in the glycolytic pathway (facilitating metabolic pathways, providing energy, and enabling material synthesis) but also affects its intracellular localization. In summary, there exists a close interrelationship between PKM2’s structural alterations, acetylation modifications, and glycolysis (12). Studies indicate that PKM2 plays a crucial role in immune metabolic reprogramming by influencing metabolic shifts, particularly promoting cells to transition from oxidative phosphorylation to anaerobic glycolysis (known as the Warburg effect), thus modulating the inflammatory levels (12, 13). Immunometabolic regulation by PKM2 can stimulate the release of a multitude of pro-inflammatory cytokines, resulting in an excessive inflammatory response (14). Additionally, PKM2 has been found to play a significant role in the development of certain inflammatory diseases, and its regulation can alleviate conditions like sepsis through multiple pathways (13, 15). PKM2-mediated immune metabolic reprogramming promotes the release of numerous pro-inflammatory cytokines, triggering an inflammatory response (16). Consequently, PKM2 is considered a potential therapeutic target for inflammatory diseases associated with cytokine storms. It modulates cellular metabolic patterns by regulating glycolysis and immune metabolism, thereby influencing inflammatory levels (14).

Our study aimed to assess the relationship and mechanisms among the SENP1-SIRT3 axis, glycolysis, and inflammation, as well as to elucidate how SENP1 contributed to protecting the host from inflammation and damage induced by *L. monocytogenes* infection.

## RESULTS

### *L. monocytogenes* inhibits inflammation by modulating glucose metabolism levels

Considering that glycolysis is a crucial metabolic pathway that regulates inflammatory responses (3). Hence, we aimed to investigate whether the inflammation induced by *L. monocytogenes* is mediated through the regulation of glycolysis.

**Fig 1:**
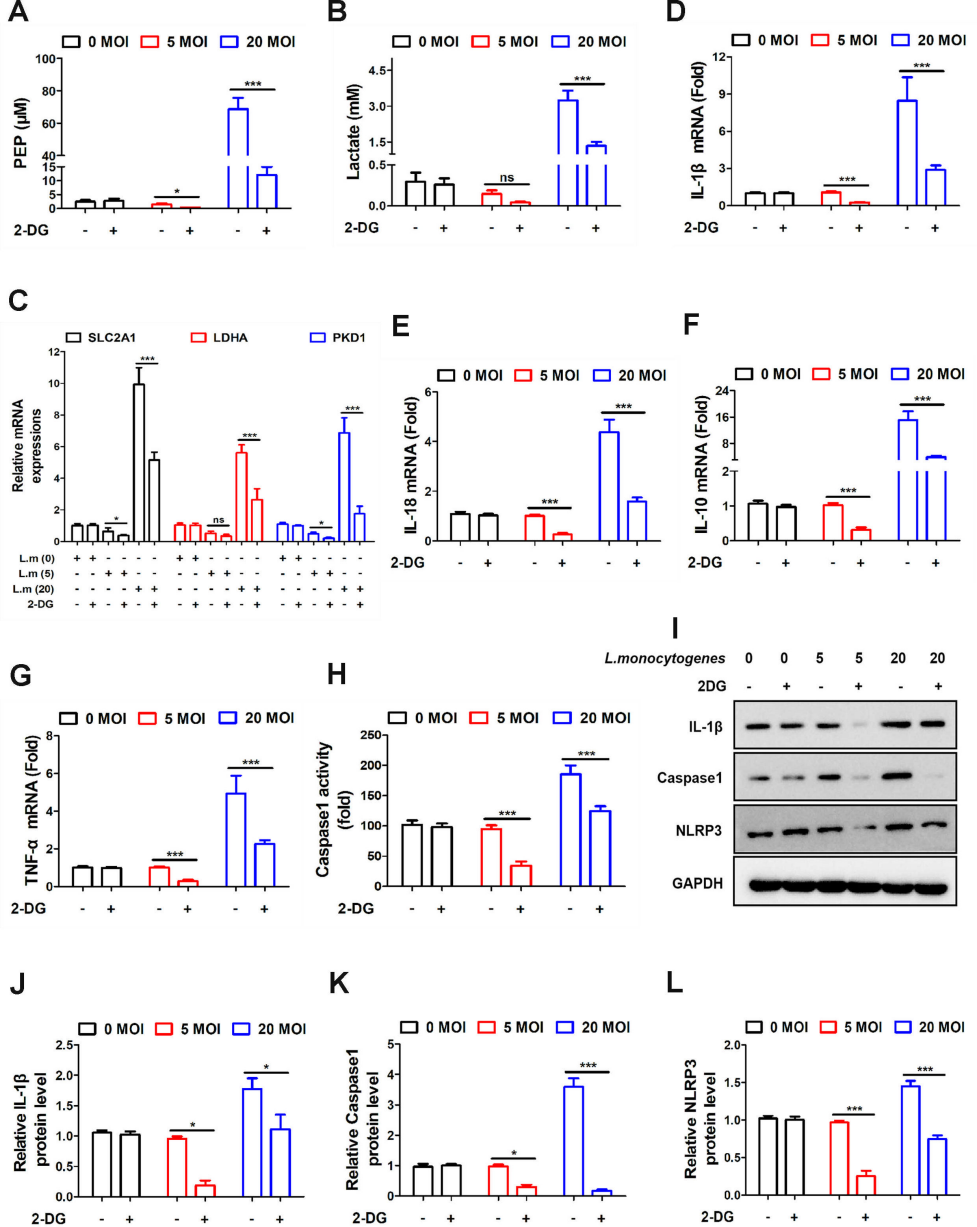
*L. monocytogenes* inhibits inflammation by modulating glucose metabolism levels. (**A**) Caco-2 cells were pre-treated with 2-DG (0.5 mM) for 2 h, and then treated with *L. monocytogenes* (*Lm*) (MOI = 0, 5, 20) for 12 h. The glycolytic activity was examined by the glycolysis assay kit. (**B**) Caco-2 cells were infected with *L. monocytogenes* (MOI = 0, 5, 20), and then the lactate levels were measured by the lactate detection kit. (**C**) 2-DG-pre-treated cells were infected with *L. monocytogenes* (MOI = 0, 5, 20) for 12 h. The expression levels of SLC2A1, LDHA, and PKD1 genes were determined by real-time quantitative PCR in each cell group. (**D–G**) The expression levels of IL-1β, IL-18, IL-10, and TNF-α were assessed by real-time quantitative PCR in different cell groups. (**H**) The caspase-1 activity assay kit was used to evaluate the activity of caspase-1. (**I**) Caco-2 cells were treated with 0.5 mM 2-DG for 2 h, followed by treatment with or without *Lm* (MOI = 0, 5, 20) for 12 h, and IL-1β, caspase-1, and NLRP3 expressions were analyzed by western blotting. (**J–L**) Statistical results for panel **I**. Data shown in panels A–H and J–L were analyzed by two-way analysis of variance (ANOVA). The blots represented three independent experiments. All data are presented as the mean ± SEM of *n* = 6. ***P < 0.001; *P < 0.05; ns, no significance.

In this study, we selected two doses of *L. monocytogenes* (5 and 20) based on a preliminary dose-response experiment, in which concentrations of 5, 10, 15, and 20 were tested, revealing a dose-dependent effect on inflammation. The lower dose (5) was used to explore the inflammatory response under conditions mimicking a mild infection, whereas the higher dose (20) was chosen to study more severe infection conditions. Next, these two doses (5 and 20) were used to further investigate inflammatory mechanisms at different exposure levels. In Fig. 1A and B, we discovered that *L. monocytogenes* (MOI = 20) can enhance glycolysis levels (column 5). 2-Deoxy-D-glucose (2-DG) is a glucose analog and glycolysis inhibitor (17). Its application resulted in a significant inhibition of glycolysis in both infection groups (Fig. 1A and B, column 4 and column 6). SLC2A1, LDHA, and PKD1 are genes related to glycolysis, playing crucial roles in transport and metabolism (18–20). Therefore, these genes can serve as indicators of glycolytic activity. In Fig. 1C, the levels of glycolysis-related genes were decreased with *L. monocytogenes* (MOI = 5) infection, while they were increased with *L. monocytogenes* (MOI = 20) infection. Simultaneously, 2-DG inhibited the glycolytic levels induced by *L. monocytogenes* infection. IL-1β, IL-18, IL-10, and TNF-α represent classical pro-inflammatory cytokines (21). These cytokines play a crucial role in activating various immune cells and promoting inflammation (22). As shown in Fig. 1D through G, the expression levels of IL-1β, IL-18, IL-10, and TNF-α were increased under the infection of *L. monocytogenes* (MOI = 20). Interestingly, the levels of IL-1β, IL-18, IL-10, and TNF-α were reduced by 2-DG treatment. This indicates that inhibiting glycolysis can suppress inflammation. The expression levels of IL-1β, as well as the inflammation-associated genes NLRP3 and caspase-1, were significantly reduced by 2-DG treatment, as predicted (Fig. 1H through L). From the above results, it can be inferred that the inflammation induced by *L. monocytogenes* was achieved through the modulation of glycolysis.

### The SENP1-SIRT3 axis is activated during *L. monocytogenes* infection

**Fig 2:**
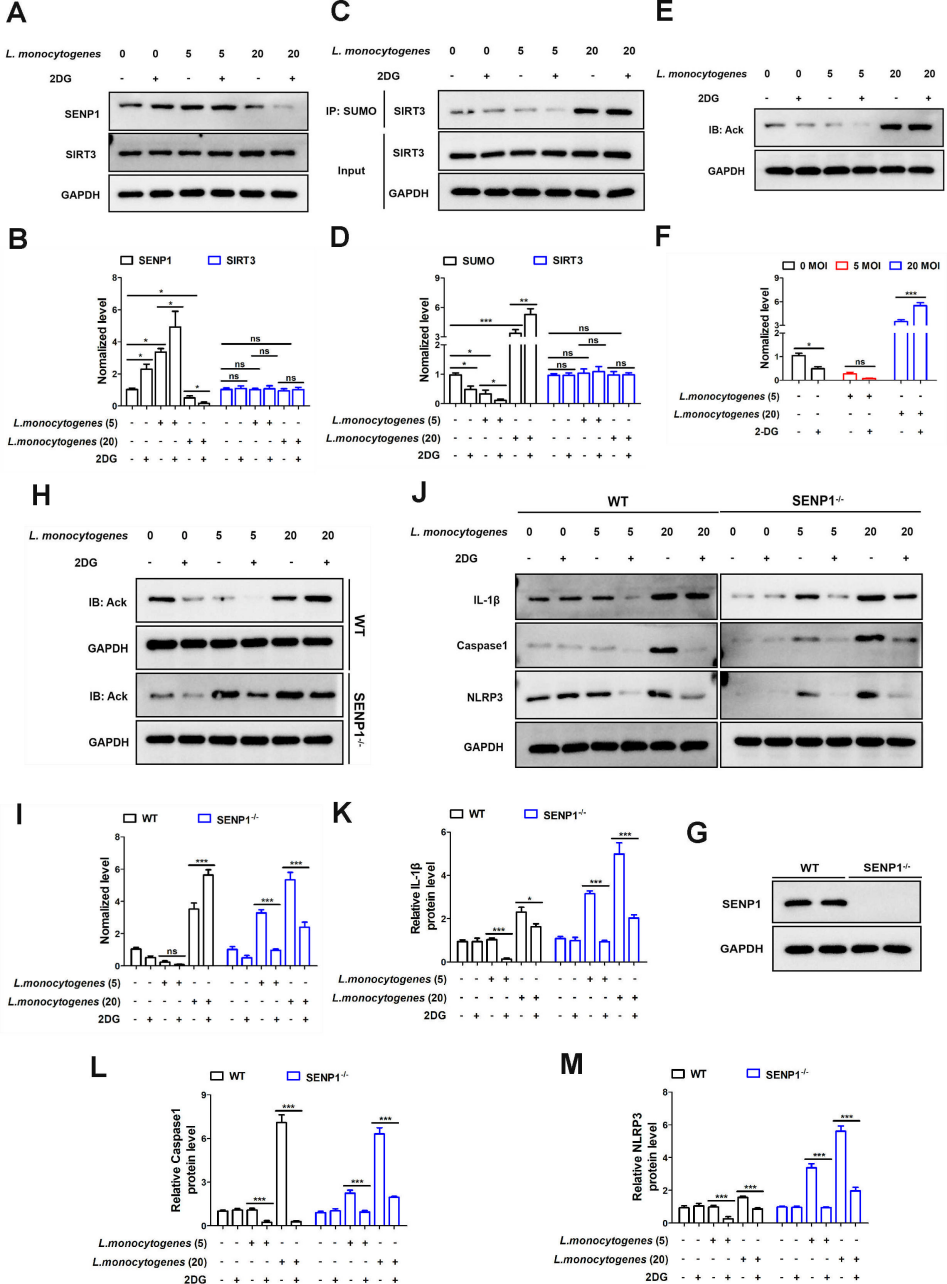
The SENP1-SIRT3 axis is activated during *L. monocytogenes* infection. (**A and B**) Caco-2 cells were treated with 0.5 mM 2-DG for 2 h, followed by treatment with or without *L. monocytogenes* (MOI = 0, 5, 20) for 12 h, and SENP1 and SIRT3 expressions were analyzed by western blotting. (**C and D**) Caco-2 cells were treated with 0.5 mM 2-DG for 2 h, followed by treatment with or without *L. monocytogenes* (MOI = 0, 5, 20) for 12 h. The SUMOylation of SIRT3 was detected by Co-IP. (**E and F**) Caco-2 cells were treated with 0.5 mM 2-DG for 2 h, followed by treatment with or without *L. monocytogenes* (MOI = 0, 5, 20) for 12 h. The global acetylation of cell total proteins was measured by immunoblotting analysis. (**G**) The SENP1 gene knockout in Caco-2 cells was confirmed by western blotting. (**H and I**) Cells (WT, SENP1^−/−^) were treated with 0.5 mM 2-DG for 2 h, followed by treatment with or without *L. monocytogenes* (MOI = 0, 5, 20) for 12 h. The global acetylation of cell total proteins was measured by immunoblotting analysis. (**J**) Cells (WT, SENP1^−/−^) were treated with 0.5 mM 2-DG for 2 h, followed by treatment with or without *L. monocytogenes* (MOI = 0, 5, 20) for 12 h, and then the IL-1β, caspase-1, and NLRP3 expressions were analyzed by western blotting. (**K–M**) Statistical results for panel **J**. The blots represented three independent experiments. The quantifications of the western blots were analyzed by two-way ANOVA.

Interestingly, it was observed that *L. monocytogenes* (MOI = 5) did not activate inflammation (Fig. 1D through I, column 3), whereas 2-DG reduced the expression of inflammatory factors (Fig. 1D through I, column 4). Therefore, we hypothesize that a particular gene plays a role in protecting the organism from bacterial invasion. The SENP1-SIRT3 axis is a widely studied cellular signaling pathway. Next, we further investigate the role of the SENP1-SIRT3 axis in metabolic reprogramming induced by *L. monocytogenes*. In the control group of aseptic infection, we observed increased expression of SENP1 in cells treated with 2-DG (Fig. 2A, lane 2). Following *L. monocytogenes* (MOI = 5) infection, SENP1 expression was further elevated (Fig. 2A, lane 3). And more, the expression of SENP1 was significantly enhanced by 2-DG (Fig. 2A, lane 4). Interestingly, when the infection dose was increased to an MOI of 20, SENP1 expression decreased (Fig. 2A, lane 5). Additionally, SENP1 expression was further reduced when cells were treated with 2-DG (Fig. 2A, lane 6). These results indicated that SENP1 may play a critical role in regulating inflammation in *L. monocytogenes*. Then, we found that the expression of SIRT3 remained unaffected with different treatments (Fig. 2A and B). Meanwhile, an increase in SENP1 led to a reduction in SUMOylated SIRT3 (Fig. 2C and D). This further revealed the activation of the SENP1-SIRT3 axis in *L. monocytogenes* infection.

Furthermore, desumoylated SIRT3 was activated and promoted mitochondrial deacetylation, as evidenced by the results in Fig. 2E and F, which align with the changes in SIRT3 SUMOylation levels shown in Fig. 2C. Subsequently, we generated SENP1-knockout Caco-2 cells (SENP1^−/−^). The knockout efficiency was validated by western blotting (Fig. 2G). In Fig. 2H and I, we observed an increase in acetylation levels in SENP1^−/−^ cells with *L. monocytogenes* treatment (MOI = 5), and this effect was eliminated by 2-DG (lane 3, lane 4). Similar results were also observed with higher-dose *L. monocytogenes* (MOI = 20) infection (lane 5, lane 6).

Interestingly, in the SENP1^−/−^ cells, *L. monocytogenes* (MOI = 5) enhanced the inflammatory response, which was effectively alleviated by 2-DG (Fig. 2J through M).

These results suggested that SENP1 plays a protective role in the inflammatory response induced by *L. monocytogenes* (MOI = 5).

### The SENP1-SIRT3 axis suppresses inflammation by inhibiting glycolysis

**Fig 3:**
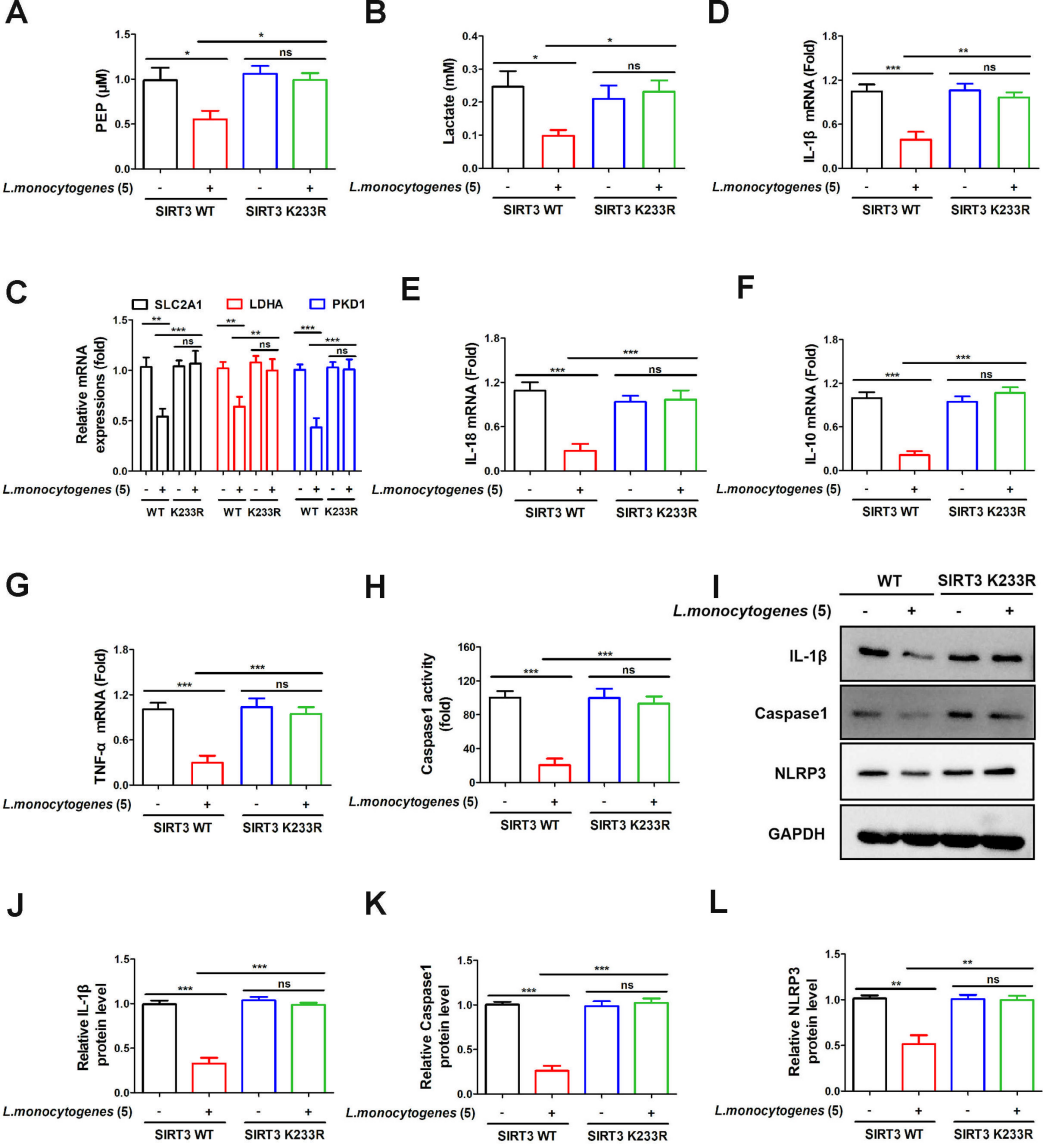
The SENP1-SIRT3 axis suppresses inflammation by inhibiting glycolysis. (**A**) Caco-2 cells (SIRT3 WT, SIRT3 K233R) were treated with or without *L. monocytogenes* (MOI = 5) for 12 h. Glycolytic activity was measured using the glycolysis assay kit. (**B**) Caco-2 cells (SIRT3 WT, SIRT3-K233R) were infected with or without *L. monocytogenes* (MOI = 5) for 12 h, and then the lactate levels were measured by the lactate detection kit. (**C**) Caco-2 cells (SIRT3 WT, SIRT3-K233R) were infected with or without *L. monocytogenes* (MOI = 5) for 12 h. The expression levels of SLC2A1, LDHA, and PKD1 genes were determined by real-time quantitative PCR in each cell group. (**D–G**) WT and SIRT3-K233R Caco-2 cells were infected with or without *L. monocytogenes* (MOI = 5) for 12 h, and then the expression levels of IL-1β, IL-18, IL-10, and TNFα were assessed by real-time quantitative PCR in different cell groups. (**H**) WT and SIRT3-K233R Caco-2 cells were infected with or without *L. monocytogenes* (MOI = 5) for 12 h. The caspase-1 activity was assessed. (**I**) WWT and SIRT3-K233R Caco-2 cells were infected with or without *L. monocytogenes* (MOI = 5) for 12 h, and then the IL-1β, caspase-1, and NLRP3 expressions were analyzed by western blotting. (**J–L**) Statistical results for panel **I**. Data shown in panels A, B, D–H, and J–K were analyzed by one-way ANOVA. Data shown in panel C was analyzed by two-way ANOVA. The blots represented three independent experiments. All data are presented as the mean ± SEM of *n* = 6. ***P < 0.001; **P < 0.01; *P < 0.05;, ns, no significance.

Next, we sought to investigate the influence of the SENP1-SIRT3 axis on glycolysis and inflammation. We introduced a mutation in the lysine 233 residue of the SIRT3 protein to generate SIRT3 K233R Caco-2 cells. SIRT3 K223R, a SUMOylation mutant, has been reported to mimic the activation of the SENP1-SIRT3 axis (8). In our study, the expression levels of glycolytic and lactate levels, as well as glycolysis-related genes, were reduced by *L. monocytogenes* (MOI = 5) infection in wild-type (WT) cells. However, these parameters showed no difference in SIRT3 K233R cells (Fig. 3A through C). Subsequently, the expression of IL-1β, IL-18, IL-10, and TNF-α was inhibited in SIRT3 WT cells by *L. monocytogenes* (MOI = 5) treatment (Fig. 3D through G). However, upon mutating SIRT3, their expressions remained unchanged, consistent with the alterations in glycolytic levels (Fig. 3D through G). Similarly, in the SIRT3 K233R cells, the expressions of caspase-1 and NLRP3 remained unaffected by *L. monocytogenes* (MOI = 5) infection (Fig. 3H through I). Consequently, *L. monocytogenes* (MOI = 5) infection failed to induce an inflammatory response. Following this, we treated higher doses (MOI = 20) of *L. monocytogenes* in Caco-2 cells. Results demonstrated a significant increase in glycolytic and lactate levels in the WT group post-infection, along with elevated expression of glycolysis-related genes. However, there were no significant changes pre- and post-infection in the SIRT3 K233R group (Fig. S1A through C). Furthermore, there was a notable increase in the expression of inflammatory factors due to higher concentration *L. monocytogenes* (MOI = 20) infection in the WT group, yet this remained unchanged pre- and post-infection in the SIRT3 K233R group (Fig. S1D through G). Finally, in the WT group, the expressions of caspase-1 and NLRP3 were significantly increased by higher concentration *L. monocytogenes* (MOI = 20) infection, while remaining unaffected in the SIRT3 K233R group (Fig. S1H through I). Our results above suggested that the activation of the SENP1-SIRT3 axis induced by *L. monocytogenes* could mitigate inflammation by suppressing glycolysis.

### The SENP1-SIRT3 axis modulates glucose metabolism by regulating PKM2 acetylation

**Fig 4:**
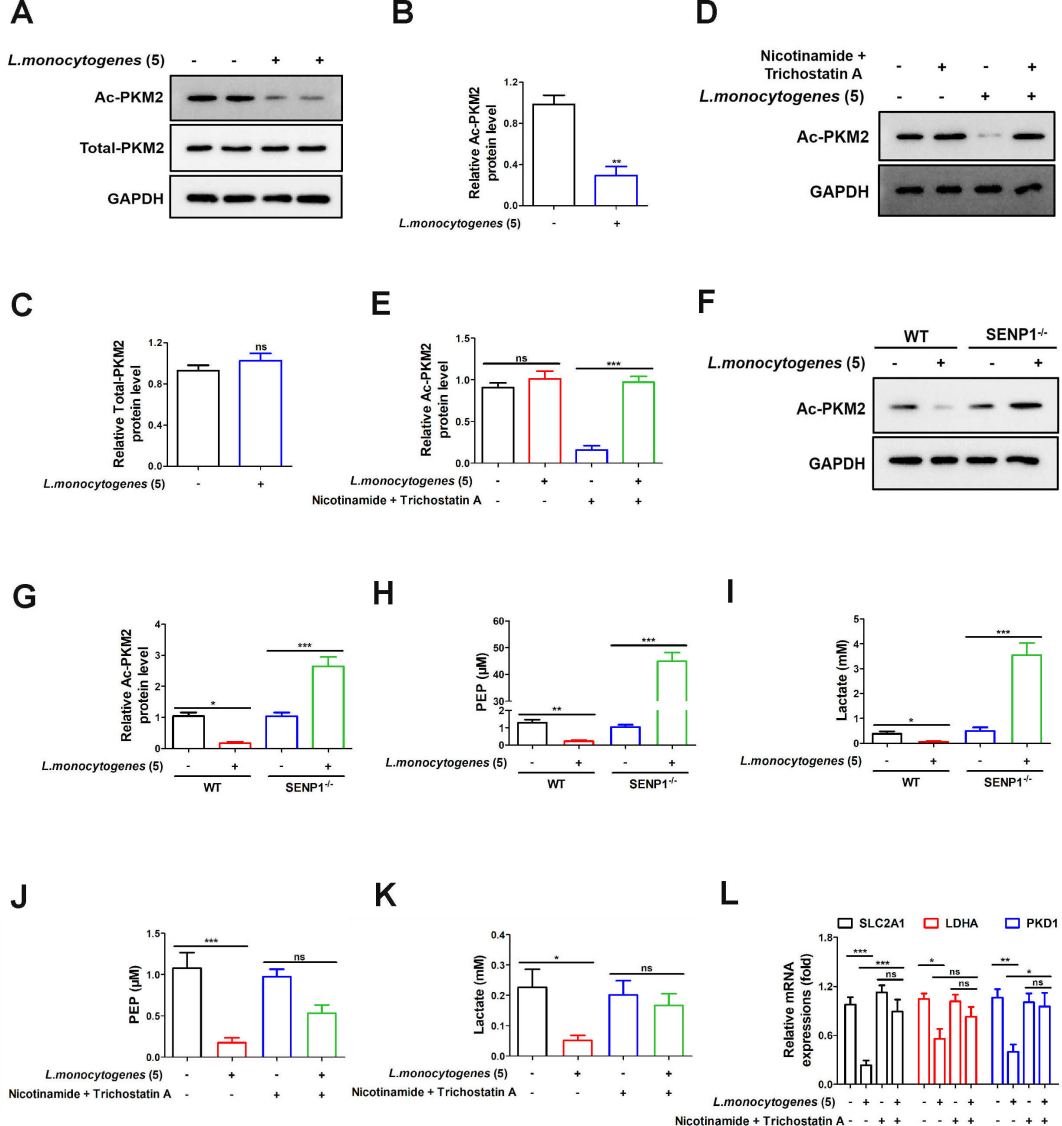
The SENP1-SIRT3 axis modulates glucose metabolism by regulating PKM2 acetylation. (**A–C**) Caco-2 cells were infected with or without *L. monocytogenes* (MOI = 5) for 12 h, and then the acetylation level of PKM2 was assessed by immunoblotting analysis. (**D and E**) Caco-2 cells were treated with nicotinamide and trichostatin A for 2 h, followed by treatment with or without *L. monocytogenes* (MOI = 5) for 12 h, and then the acetylation level of PKM2 was assessed by immunoblotting analysis. (**F and G**) Caco-2 cells (WT, SENP1^−/−^) were infected with or without *L. monocytogenes* (MOI = 5) for 12 h, and then the acetylation level of PKM2 was assessed by immunoblotting analysis. (**H**) Caco-2 cells (WT, SENP1^−/−^) were infected with or without *L. monocytogenes* (MOI = 5) for 12 h, and then the glycolytic activity was examined by the glycolysis assay kit. (**I**) Caco-2 cells (WT, SENP1^−/−^) were infected with or without *L. monocytogenes* (MOI = 5) for 12 h, and then the lactate levels were measured by the lactate detection kit. (**J and K**) Caco-2 cells were treated with nicotinamide + trichostatin A for 2 h, followed by treatment with or without *L. monocytogenes* (MOI = 5) for 12 h, and then the glycolytic activities and lactate levels were measured. (**L**) Caco-2 cells were treated with nicotinamide (5 mM) and trichostatin A (1 µM) for 2 h, followed by treatment with or without *L. monocytogenes* (MOI = 5) for 12 h. The expression levels of SLC2A1, LDHA, and PKD1 genes were determined by real-time quantitative PCR in each cell group. Data shown in panels B and C were analyzed by *t*-test. Data shown in panels E and G–K were analyzed by one-way ANOVA. Data shown in panel L was analyzed by two-way ANOVA. The blots represented three independent experiments. All data are presented as the mean ± SEM of *n* = 6. ***P < 0.001; **P < 0.01; ns, no significance.

PKM2, an isoform of the glycolytic enzyme pyruvate kinase, has a close interrelation with its structural alterations, acetylation modifications, and glycolysis (12). In light of our earlier findings, which demonstrated an overall decrease in cellular acetylation levels and an impact on glycolysis following *L. monocytogenes* (MOI = 5) infection (Fig. 1). And then, we discovered a significant reduction in the levels of acetylated PKM2, while the overall expression level of PKM2 remained unaltered (Fig. 4A). Caco-2 cells were treated with nicotinamide (a pan-SIRT inhibitor) and trichostatin A (a histone deacetylase inhibitor) (23). Under *L. monocytogenes* (MOI = 5) infection, the expression level of acetylated PKM2 in lane 4, treated with nicotinamide and trichostatin A, was restored compared to the low-level expression observed in lane 3 (Fig. 4B). It indicated the crucial role of SIRT3 in the deacetylation process of PKM2. Next, we found a significant increase in the levels of PKM2 acetylation in SENP1^−/−^ cells by *L. monocytogenes* (MOI = 5) infection, while the WT cells exhibited a contrary trend of reduced acetylation levels (Fig. 4C). Additionally, upon re-evaluating glycolysis and lactate levels, the SENP1^−/−^ group showed a significant increase in contrast to the decreasing trend observed in the WT group (Fig. 4D and E). As shown in Fig. 4F through H, the combination of nicotinamide and trichostatin A reversed the glycolytic suppression induced by *L. monocytogenes* (MOI = 5). These findings demonstrated that the SENP1-SIRT3 axis can regulate glucose metabolism by modulating PKM2 acetylation.

### Disruption of the SENP1-SIRT3 axis aggravates inflammation

Considering the role of the SENP1-SIRT3 axis in regulating glucose metabolism and its connection to glycolytic levels affected by *Listeria*-triggered inflammation.

**Fig 5:**
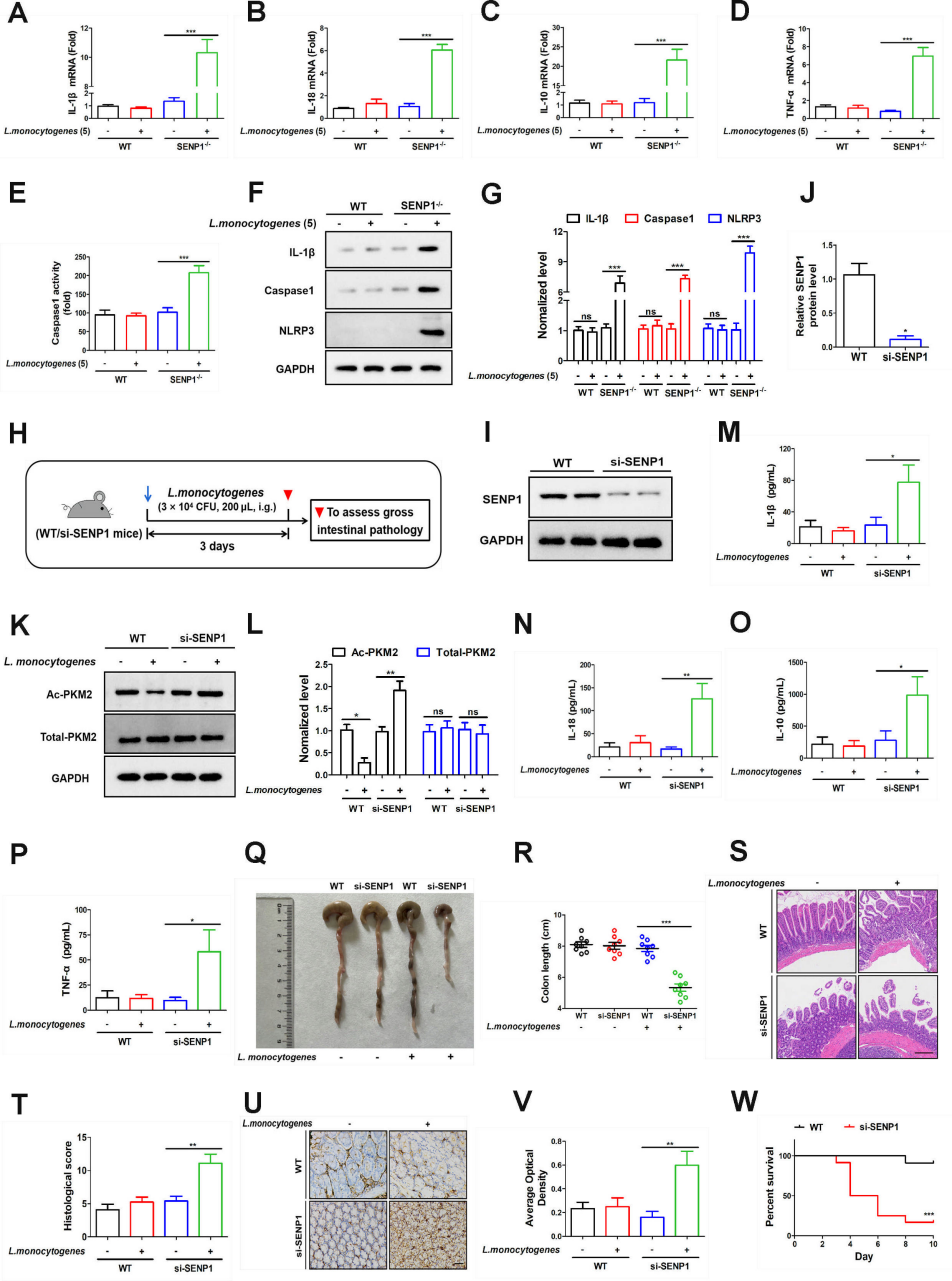
Disruption of the SENP1-SIRT3 axis aggravates inflammation. (**A–D**) Caco-2 cells (WT, SENP1^−/−^) were infected with *L. monocytogenes* (MOI = 5) for 12 h. RNA was extracted, and then the expression levels of IL-1β, IL-18, IL-10, and TNFα were assessed by real-time quantitative PCR in different cell groups. (**E**) The caspase-1 activity was assessed using a caspase-1 activity assay kit. (**F and G**) Caco-2 cells (WT, SENP1^−/−^) were infected with *L. monocytogenes* (MOI = 5) for 12 h, with respective uninfected control groups. Cell total proteins were extracted, and then the expression levels of IL-1β, caspase-1, and NLRP3 were measured by western blotting. (**H**) Schematic diagram demonstrating the study design of *in vivo* experiments. Mice (WT, si-SENP1) were treated with *L. monocytogenes* (3 × 10^4^ CFU, 200 µL, i.g.) for 3 days. Mice were euthanized, and gross intestinal pathology was assessed. (**I and J**) The SENP1 gene was knocked down in the colon. Colonic tissue proteins were extracted, and the knockdown efficiency was validated by western blotting. (**K and L**) Mice (WT, si-SENP1) were treated with *L. monocytogenes* (3 × 10^4^ CFU). Colonic tissue proteins were extracted, and then the acetylation level of PKM2 was assessed by immunoblotting analysis. (**M–P**) Mice (WT, si-SENP1) were treated with *L. monocytogenes* (3 × 10^4^ CFU). Serum was extracted, and the expression levels of IL-1β, IL-18, IL-10, and TNFα were measured by enzyme-linked immunosorbent assay (ELISA). (**Q and R**) Mice (WT, si-SENP1) were treated with *L. monocytogenes* (3 × 10^4^ CFU). Mice were euthanized, and the length of colons was measured. (**S and T**) Hematoxylin and eosin (H&E) staining analysis (**S**) and histology score (**T**) in colon tissues. Scale bar, 100 µm. (**U and V**) Immunohistochemical staining of mice colon tissue sections from WT and si-SENP1 mice for TNF-α. Tissue sections were stained with an anti-TNF-α antibody to visualize TNF-α expression. Brown staining indicates positive TNF-α expression. Scale bar, 25 µm. (**W**) Mice (WT, si-SENP1) were treated with *L. monocytogenes* (3 × 10^4^ CFU) for 10 days. Mouse survival rates were monitored. Data shown in panels A–E, M–P, R, T, and V were analyzed by one-way ANOVA. Data shown in panels G and L were analyzed by two-way ANOVA. Data shown in panel W was analyzed by the survival curve. The blots represented three independent experiments. All data are presented as the mean ± SEM of *n* = 6. ***P < 0.001.

What impact would disrupting the SENP1-SIRT3 axis have on inflammation induced by *L. monocytogenes* infection? Next, we aimed to further investigate the role of the SENP1-SIRT3 axis in the inflammation induced by *L. monocytogenes* infection. Firstly, Caco-2 cells (WT, SENP1^−/−^) were treated with *L. monocytogenes* (MOI = 5). While the WT group did not exhibit significant differences in inflammatory factors, various inflammatory markers significantly increased in the SENP1^−/−^ group (Fig. 5A through D). The deletion of SENP1 resulted in increased caspase-1 enzyme activity (Fig. 5E), confirmed by western blotting analysis showing significant upregulation in the expression levels of IL-1β, caspase-1, and NLRP3 proteins (Fig. 5F and G). These findings implied that the deletion of SENP1 exacerbated the inflammation induced by *L. monocytogenes* (MOI = 5) infection. To further delineate the role of the SENP1-SIRT3 signaling pathway in *L. monocytogenes*-mediated inflammation, we established the SENP1 knockdown mice model (Fig. 5H).

The knockdown efficiency was validated by western blotting (Fig. 5I and J).

Subsequently, we obtained mouse intestinal tissue to examine its acetylation levels.

As shown in Fig. 5K and L, we noted an increase in acetylated PKM2 expression in the colon tissues of si-SENP1 mice, while acetylated PKM2 expression decreased in WT mice (lane 4). Additionally, the levels of IL-1β, IL-18, IL-10, and TNF-α were elevated in the serum of si-SENP1 mice following treatment with *L. monocytogenes*, as determined by enzyme-linked immunosorbent assay (ELISA) (Fig. 5M through P, column 4). Meanwhile, we have also added more intestinal inflammatory markers in our study to further confirm our results using western blotting. TNF-α is a central cytokine in inflammatory reactions. Therefore, we validated the inflammatory response in mice by examining the levels of TNF-α across different treatment groups. In the mouse infection model, infection with *L. monocytogenes* (3 × 10^4^ CFU) did not result in changes in TNF-α expression (Fig. S2A and B, lane 1, lane 2). In the si-SENP1 group, infection at 3 × 10^9^ CFU exacerbated inflammation (Fig. S2A and B, lane 3, lane 4). In WT mice, we observed that *L. monocytogenes-*induced inflammation did not cause significant changes in colon length. However, in si-SENP1 mice, a notable reduction in colon length was evident (Fig. 5Q through R). Furthermore, hematoxylin and eosin (H&E) staining revealed more severe tissue injury in the colon tissues of si-SENP1 mice following *L. monocytogenes* infection (Fig. 5T). IHC analysis of mice colon tissue sections from WT and si-SENP1 mice using an anti-TNF-α antibody showed darker brown staining in the si-SENP1 group compared to WT, indicating higher TNF-α expression in si-SENP1 mice (Fig. 5U and V). Additionally, after a 10-day survival observation, *L. monocytogenes* infection resulted in substantial mortality in si-SENP1 mice, whereas over 80% of infected WT mice survived (Fig. 5W). These data indicated that the disruption of the SENP1-SIRT3 axis exacerbated *L. monocytogenes*-induced inflammation, thereby increasing mortality in mice. Collectively, these findings revealed that the SENP1-SIRT3 axis plays a crucial role in *L. monocytogenes* infection. Blocking this axis led to exacerbated inflammation.

### Enhancement of organism protection by the SENP1-SIRT3 axis

**Fig 6:**
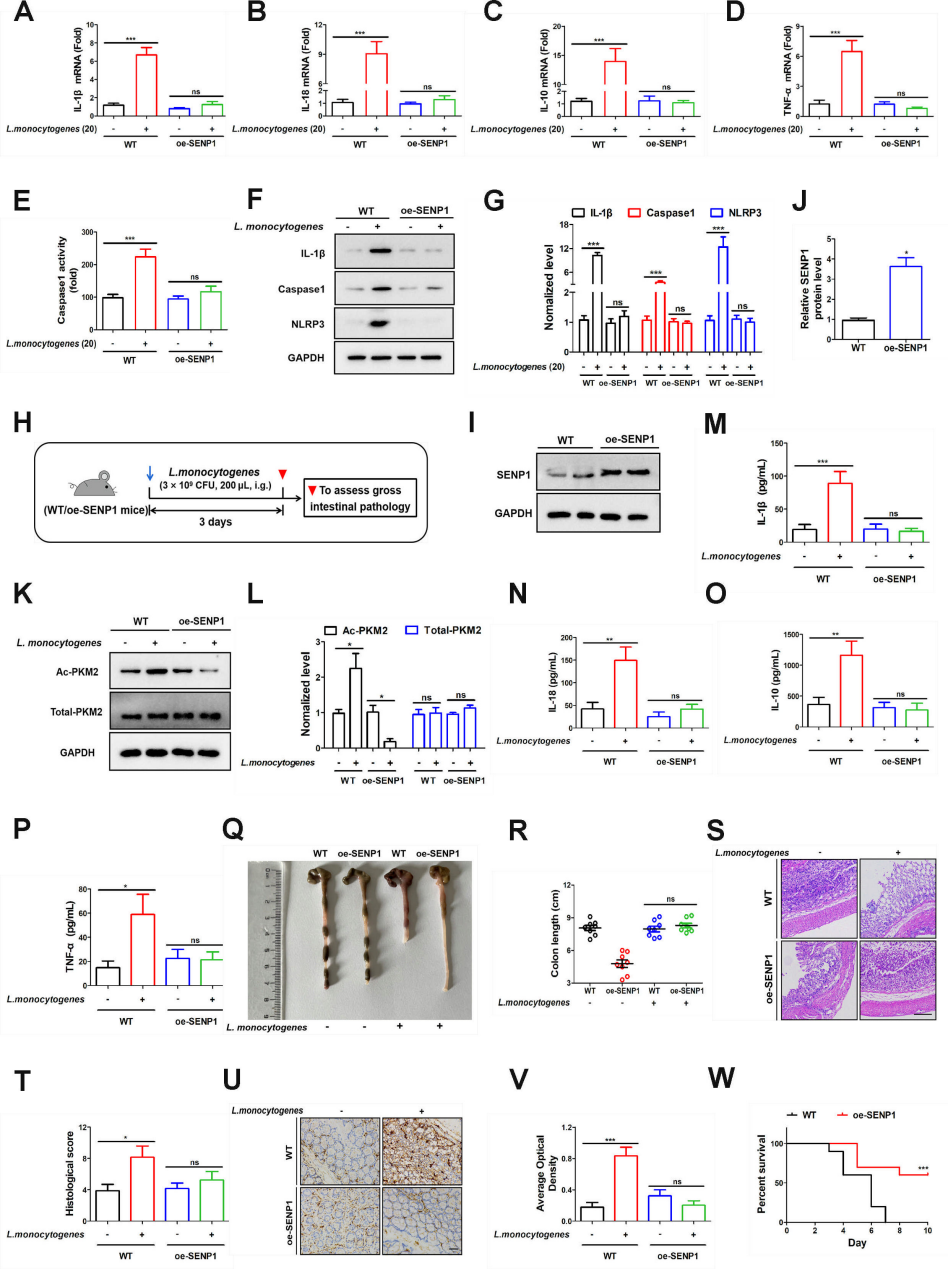
Enhancement of organism protection by the SENP1-SIRT3 axis. (**A–D**) The overexpression of SENP1 was subsequently assessed for its impact on the expression levels of IL-1β, IL-18, IL-10, and TNF-α, both in the presence and absence of *L. monocytogenes* treatment. (**E**) Caco-2 (WT, oe-SENP1) cells were infected with *L. monocytogenes* (MOI = 20) for 12 h, with respective uninfected control groups. Caspase-1 activity was assessed using a caspase-1 activity assay kit. (**F**) Caco-2 cells (WT, oe-SENP1) were infected with *L. monocytogenes* (MOI = 5) for 12 h, with respective uninfected control groups. Cell total proteins were extracted, and then the expression levels of IL-1β, caspase-1, and NLRP3 were measured by western blotting. (**G**) Statistical results for panel **F**. (**H**) Schematic diagram demonstrating the study design of *in vivo* experiments. Mice (WT, oe-SENP1) were treated with *L. monocytogenes* (3 × 10^9^ CFU) for 3 days. Mice were euthanized, and gross intestinal pathology was assessed. (**I and J**) The SENP1 gene was overexpressed in the colon. Colonic tissue proteins were extracted, and the overexpression efficiency was validated by western blotting. (**K**) Mice (WT, oe-SENP1) were treated with *L. monocytogenes* (3 × 10^9^ CFU). Colonic tissue proteins were extracted, and then the acetylation level of PKM2 was assessed by immunoblotting analysis. (**L**) Statistical results for panel **K**. (**M–P**) Mice (WT, oe-SENP1) were treated with *L. monocytogenes* (3 × 10^9^ CFU). Serum was extracted, and the expression levels of IL-1β, IL-18, IL-10, and TNF-α were measured by ELISA. (**Q and R**) Mice (WT, oe-SENP1) were treated with *L. monocytogenes* (3 × 10^9^ CFU). Mice were euthanized, and the length of colons was measured. (**S and T**) H&E staining analysis (**S**) and histology score (**T**) in colon tissues. Scale bar, 100 µm. (**U and V**) Immunohistochemical staining of mice colon tissue sections from WT and oe-SENP1 mice for TNF-α. Tissue sections were stained with an anti-TNF-α antibody to visualize TNF-α expression. Brown staining indicates positive TNF-α expression. Scale bar, 25 µm. (**W**) Mice (WT, oe-SENP1) were treated with *L. monocytogenes* (3 × 10^9^ CFU) for 10 days. Mouse survival rates were monitored. Data shown in panels A–E, M–P, R, T, and V were analyzed by one-way ANOVA. Data shown in panels G and L were analyzed by two-way ANOVA. Data shown in panel W was analyzed by the survival curve. The blots represented three independent experiments. All data are presented as the mean ± SEM of *n* = 6. ***P < 0.001; ns, no significance.

The earlier experimental outcomes shed light on the pivotal role of the SENP1-SIRT3 axis in the onset of *L. monocytogenes*-mediated inflammation. We postulated that the SENP1-SIRT3 axis exerts a favorable role in this mechanism by functioning as a positive modulator of organismal defense. We overexpressed SENP1 in Caco-2 cells to establish the oe-SENP1 cell model. Cells were infected with *L. monocytogenes* (MOI = 20), inflammatory factors (IL-1β, IL-18, IL-10, TNF-α) exhibiting a significant upregulation in the WT group (Fig. 6A through D, column 2). In line with our speculation, the overexpression of SENP1 effectively reversed the expression levels of various inflammatory factors. Moreover, the caspase-1 enzyme activity was also increased in the WT group in Fig. 6E. Additionally, similar results were observed in the expression levels of IL-1β, caspase-1, and NLRP3 proteins (Fig. 6F and G). We established the SENP1 overexpression mice model to further investigate (Fig. 6H). The overexpression efficiency was validated by western blotting (Fig. 6I and J).

In this model, we also obtained mouse intestinal tissue to examine its acetylation levels. In Fig. 6K and L, we noted a decrease in acetylated PKM2 expression in the colon tissues of oe-SENP1 mice, while acetylated PKM2 expression increased in WT mice (lane 4). More importantly, the levels of IL-1β, IL-18, IL-10, and TNF-α showed no significant change in the serum of oe-SENP1 mice following treatment with *L. monocytogenes*, as determined by ELISA (Fig. 6M through P, column 4). In the mouse infection model, infection with *L. monocytogenes* (3 × 10^9^ CFU) increased TNF-α expression levels (Fig. S2C and D, lane 1, lane 2). In the oe-SENP1 group, mice were protected from inflammation caused by *L. monocytogenes* (3 × 10^9^ CFU) infection (Fig. S2C and D, lane 3, lane 4). The colons of oe-SENP1 mice were notably longer than those of WT mice and similar to the uninfected group when treated with *L. monocytogenes* (Fig. 6Q and R). Furthermore, H&E staining also demonstrated that overexpression of SENP1 in mice (oe-SENP1) exerted a protective effect on colon tissues following *L. monocytogenes* infection (Fig. 6T). Immunohistochemical staining for TNF-α on colon tissue sections from WT and oe-SENP1 mice revealed that overexpression of SENP1 significantly attenuated inflammation (Fig. 6U and V). When observing the survival rate of infected mice within 10 days, all WT mice died within 7 days, whereas the survival rate of oe-SENP1 mice was maintained at around 60% by day 10 (Fig. 6W). This implied that SENP1 can play a role in alleviating inflammation. In summary, the overexpression of SENP1 enhanced the activation of the SENP1-SIRT3 axis, which subsequently exerts its effects, enhancing the resilience of mice against Listeria infection and significantly strengthening the organism’s defensive capacities.

## DISCUSSION

**Fig 7:**
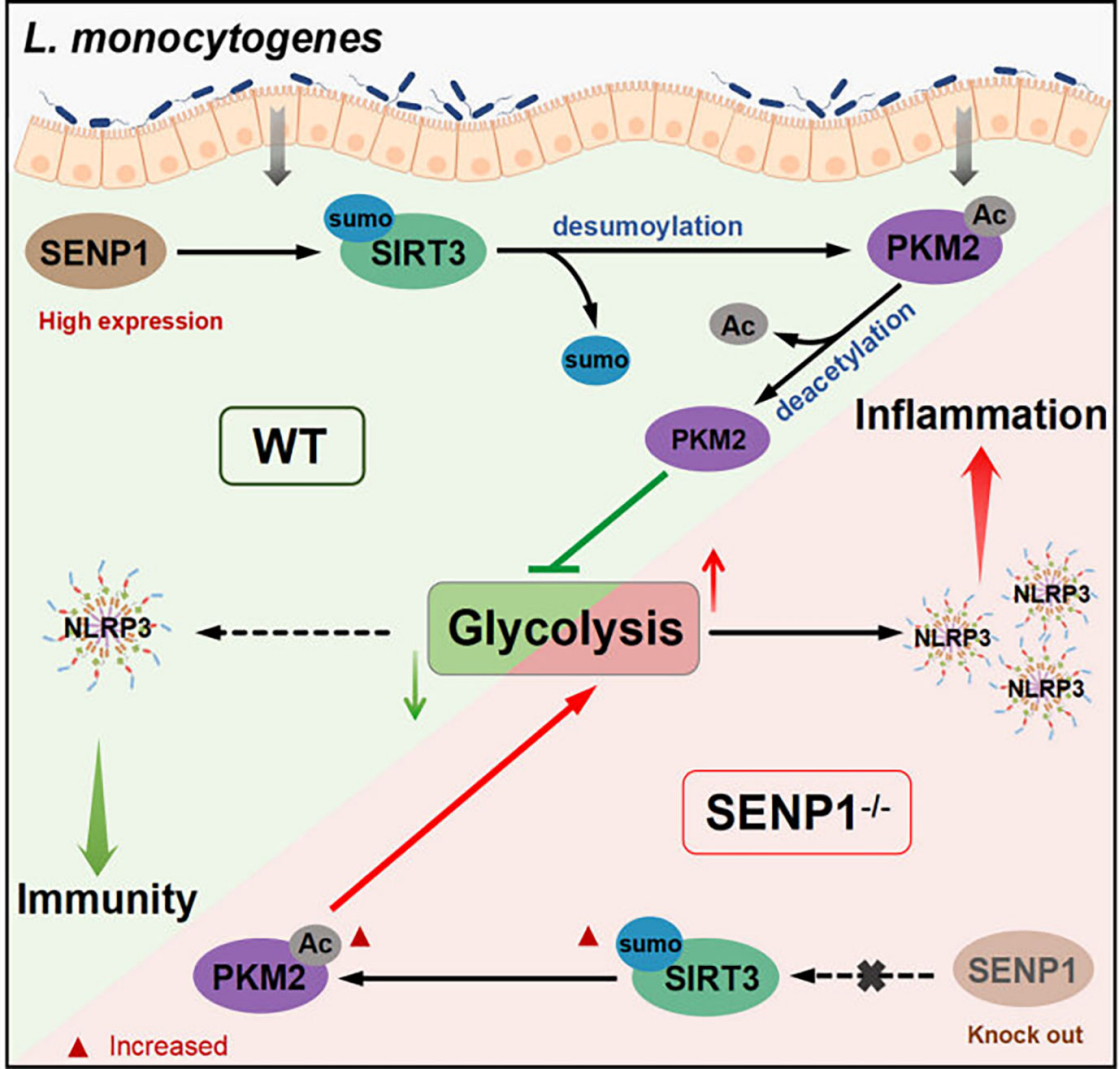
Schematic representation of *L. monocytogenes*-induced inflammation by regulating the SENP1-SIRT3 axis.

In our study, we found that SENP1-SIRT3 signaling can attenuate *Listeria*-induced inflammation by regulating glycolysis. Mechanistically, upon Listeria infection, cells activate increased expression of mitochondrial SENP1 protein. Concurrently, SENP1 mediated SIRT3 desumoylation, enhancing SIRT3 catalytic activity, thereby enhancing the deacetylation level of PKM2 and reducing PKM2 catalytic activity, ultimately inhibiting glycolysis to achieve anti-inflammatory effects (Fig. 7). This mechanism may also be relevant to infections caused by other pathogenic microorganisms. In our study, we focused on *L. monocytogenes*, but we believe that the protective role of the SENP1-SIRT3 axis could extend beyond this specific pathogen. Firstly, both SENP1 and SIRT3 play essential roles in regulating immune responses and metabolic processes. SENP1, a key enzyme in the SUMOylation pathway, modulates inflammation by regulating the SUMOylation of NF-κB essential modulator (NEMO), which in turn regulates NF-κB activity (24, 25). NF-κB is a critical factor in immune signaling (26, 27). SIRT3, a mitochondrial deacetylase, is involved in controlling cellular metabolism and oxidative stress responses, both of which are crucial during bacterial infections (28). These two proteins are likely to have conserved functions across various bacterial infections, influencing both immune regulation and metabolic adaptations. Secondly, the SENP1-SIRT3 axis plays a critical role in regulating T cell development and macrophage polarization (9, 29), both fundamental to immune responses. Therefore, targeting the SENP1-SIRT3 pathway may offer therapeutic benefits in other bacterial infections by modulating immune and metabolic processes. However, while we hypothesize that the SENP1-SIRT3 axis may play a similar protective role in other infections, additional studies using diverse bacterial models are required to validate this hypothesis and further explore its applicability in different infection contexts.

In our study, the regulation of metabolic reprogramming on *Listeria*-induced inflammatory responses was a significant discovery (Fig. 1). As we know, glucose serves as the primary carbon source for most microbes, and bacteria generate various metabolic byproducts and energy through pathways such as glycolysis, the TCA cycle, and the electron transport chain to facilitate their growth (30). Following infection of host cells, *L. monocytogenes* can manipulate the host cell’s metabolic pathways to induce the provision of more glucose sources for its own utilization, thereby promoting its growth and replication. *L. monocytogenes* metabolizes glucose into pyruvic acid through the glycolytic pathway, generating ATP through substrate-level phosphorylation (31). As we know, when cells are stimulated externally, NLRP3 aggregates to form the inflammasome, activating the caspase-1 enzyme and cleaving IL-1β and IL-18 precursor proteins, transforming them into mature cytokines that are released into the extracellular space, thus triggering an inflammatory response (32). In our study, the application of 2-DG to restrict glycolysis inhibited the intracellular growth of *L. monocytogenes* and reduced pro-inflammatory factors (IL-1β, IL-18, IL-10, and TNF-α) accordingly (Fig. 1D through G). More importantly, during the process of metabolic reprogramming regulated by *L. monocytogenes*, we have found a crucial role played by the SENP1-SIRT3 axis.

In the SENP1-SIRT3 axis, SENP1 can remove SUMO modifications from SIRT3, thereby increasing SIRT3’s activity. SIRT3, in turn, can remove acetyl modifications from various proteins, including multiple metabolism-related proteins and transcription factors, affecting their stability, subcellular localization, interactions, and functions (8, 33).

In earlier research, Tianshi Wang et al. discovered that fasting in mice decreases mitochondrial SUMOylation levels, with SIRT3 deSUMOylation being a rapid response during fasting. They found that fasting induces the translocation of SENP1 into mitochondria to activate SIRT3 deSUMOylation (8). Therefore, this study primarily focuses on the relationship between the SENP1-SIRT3 axis, glycolysis, and inflammation. Notably, there is evidence suggesting that endothelial SIRT3 regulates the metabolic switch between glycolysis and mitochondrial respiration (34). PKM2 is a key regulatory factor in metabolic processes, and its acetylation modifications can influence PKM2’s activity and function (35). Studies indicate that acetylation modifications of PKM2 can enhance tumor cell proliferation and transformation, augmenting their glucose uptake and utilization, thereby providing substrates necessary for energy and biosynthesis (36). In our study, we observed that infection with *L. monocytogenes* can induce cellular PKM2 deacetylation (Fig. 4A through C). Interestingly, deficiency of SENP1 inhibits PKM2 deacetylation mediated by the SENP1-SIRT3 signaling pathway, inhibiting the glycolytic process (Fig. 4D through L). This suggested that the SENP1-SIRT3 signaling pathway’s facilitation of PKM2 deacetylation is a critical regulatory mechanism for inhibiting glycolysis and the subsequent increase of caspase-1 and NLRP3-mediated inflammatory factors during *L. monocytogenes* infection. However, our study is not devoid of limitations. We have yet to undertake further investigation into the specific protein sites where the SENP1-SIRT3 axis regulated the deacetylation of PKM2.

In summary, our study demonstrated that SENP1 acts as a protective factor against Listeria-induced inflammation by promoting SIRT3 de-SUMOylation and subsequent metabolic reprogramming. Moreover, the SENP1-SIRT3 axis reversely mediated the deacetylation of PKM2, inhibiting the progression of glycolysis. Therefore, the SENP1-SIRT3 axis served not only as an essential signaling pathway for regulating mitochondrial metabolic responses to metabolic stress but also responds to bacterial invasion and plays a protective role in the organism. Our findings provide a basis for further research into targeting the SENP1-SIRT3 signaling pathway for the treatment of bacterial infections.

## MATERIALS AND METHODS

### Mice

C57BL/6 male mice (6-8 weeks old) were purchased from Hunan SJA Laboratory Animal Co, Ltd. (Hunan, China). Mice were treated with *L. monocytogenes* (3 × 10^4^/3 × 10^9^ CFU, 200 μL, i.g.) for 3 days. To assess gross intestinal pathology, animals were euthanized, and the length of colons were measured.

### Knockdown mouse model

*In vivo* gene knockdown was performed as previously described (37). Briefly, siRNA (40 µg) was combined with the in vivo-jetPEI delivery reagent (Polyplus-transfection, NY, USA) in a 5% glucose solution (N/P ratio = 8). The solution was mixed and incubated at room temperature for 30 min and was then intraperitoneally injected into mice.

The siRNA sequences are listed as follows: si-SENP1: AGAAAUUGAUGAUCUCAUCAU.

Knockdown efficiency in colonic tissue was detected by western blotting.

### Generation of SENP1 overexpression in mice

SENP1 was overexpressed in the colons of mice. The adeno-associated virus 2/9 vector overexpressing SENP1 or the control vector was injected into the colon of the mice, with the FABP1 promoter and SENP1 sequences listed in Table S1.

### Cell culture and transfection

Caco-2 cells were obtained from the American Type Culture Collection (ATCC; Manassas, VA). Cells were cultured in DMEM medium (Gibco, San Jose, CA, USA) containing 10% (vol/vol) fetal bovine serum (FBS) (Gibco). All cells were kept in a humidified incubator at 37°C with CO_2_.

Cells were grown to 70% confluence before transfection. Cells were transfected (Lipofectamine 3000 reagent, Thermo Fisher Scientific) based on the manufacturer’s protocols.

### RNA extraction

According to the manufacturer’s instructions, total RNA was extracted with a Trizol (Thermo Fisher Scientific) reagent. Quantitative PCR experiments were performed using a SuperScript III One-Step RT-PCR kit (Thermo Fisher Scientific). Briefly, this procedure included 30 s of preincubation at 95°C, 40 cycles of denaturation at 95°C for 5 s, and annealing for 30 s at 60°C. The data were expressed using 2^−ΔΔCt^ method. The primers are listed in Table S1.

### Biochemical assays

Cells were placed into 6-well plates at a density of 2 × 10^5^/well and cultured at 37°C for 48 h, then the lactate production of cells was examined using Lactate Colorimetric Assay Kits (Abcam, Cambridge, MA, USA, ab65331). The supernatant was collected from cells kept in an FBS-free medium for hours and used for the measurement of lactate production. The reaction mixture was incubated for 30 min at room temperature in the dark. The lactate levels were measured at 450 nm in a microplate reader. The phosphoenolpyruvic acid production of cells was examined using the phosphoenolpyruvic acid (PEP) assay kit (Abcam, Cambridge, MA, USA, ab204713). The supernatant was collected from cells kept in an FBS-free medium for hours and used for the measurement of lactate production. A total of 50 µL of the reaction mixture was prepared and added to each well containing the standard curve and test samples. The reaction mixture was incubated for 1 h at room temperature and protected from light. The phosphoenolpyruvic acid levels were measured at 570 nm in a microplate reader.

### Caspase-1 activity analysis

The caspase-1 activity was measured by using a caspase-1 activity assay kit (Beyotime, Shanghai, China). In brief, 50 µg protein from 2 × 10^6^ Caco-2 cell lysates was added to a reaction buffer containing Ac-YVAD-ρNA (2 mM) and incubated for 2 h at 37°C. The absorbance was measured at 405 nm with a microplate reader, and the caspase-1 activity was normalized for the total proteins of cell lysates.

### Western blotting

Cell lysates were prepared using RIPA buffer (Thermo Fisher Scientific) containing a mixture of protease and phosphatase inhibitors. Protein concentration was quantified using the BCA assay kit (Beyotime, P0010). Electrophoresis on 10% sodium dodecyl sulfate-polyacrylamide gel was used to separate protein extracts, and the results were then transferred to a polyvinylidene fluoride membrane. Following incubation with primary antibodies at 4°C overnight and secondary antibodies at room temperature, membranes were blocked with 5% milk at room temperature for 2 h. Using an enhanced chemiluminescence (ECL) technique, immuno-labeled proteins were identified (ChemiScope 6100). Quantitative analysis was conducted using ImageJ software. Primary antibodies in this study used for immunoblotting are listed in Table S2.

### Immunoblot and immunoprecipitation

Caco-2 cells were washed twice with phosphate buffered saline (PBS) and then lysed with radioimmunoprecipitation assay (RIPA) lysis buffer. The scraped cells were transferred to Eppendorf tubes and centrifuged at 13,000 × *g* for 15 min. The supernatants were immediately transferred to new tubes, and the total protein was quantified using a BCA protein assay kit. Protein (500 µg) was transferred to a new tube, and 50% protein A/G-agarose was added. The antigen-antibody complex was incubated on a rotary shaker for 12 h, followed by centrifugation at 13,000 × *g* for 5 s. The precipitate was washed three times with pre-chilled washing buffer, while the supernatant was collected for analysis using SDS-PAGE and western blotting methods.

### Generation of CRISPR-Cas9-based knockout cells

Next, using the CRISPR-Cas9 method, SENP1 KO cells were produced. sgRNAs were designed (SENP1 KO-sgRNA: TATAATCCAAGCTATTACTC) and ligated into the pSpCas9 (BB)-2A-Puro (PX459) plasmid following Bbs I digestion. The recombinant was then transfected into Caco-2 cells by using Lipo 3000 Transfection Reagent (Invitrogen). Forty-eight hours after transfection, puromycin (3 µg/mL) was used for the screening for 7 days to obtain the cell pool. Total cell protein was extracted, and western blotting was used to detect expressions of SENP1.

### Serum cytokine analysis

Mouse serum cytokine levels were measured using an ELISA method. IL-1β (BMS6002), IL-18 (BMS618-3), 1L-10 (BMS614), and TNF-α (BMS607-3) ELISA kits were purchased from e-Biosciences (Thermo Fisher Scientific). In brief, serum was isolated from blood sampled by eyeball extirpation and stored at −80°C until analysis. Samples were thawed on ice and diluted appropriately with assay buffer. Diluted samples and cytokine standards were added to ELISA plates and incubated. After washing, detection antibody and substrate solutions were added sequentially. The colorimetric reaction was stopped, and absorbance was measured at 450 nm. Cytokine concentrations were determined from standard curves.

### Histopathological evaluation

Mice colonic tissue was embedded in paraffin and sectioned after fixing in paraformaldehyde for 24 h. Hematoxylin and eosin (H&E) staining was used for histological analysis. All histological images were captured through the panoramic scanner. Inflammation was scored blindly using four parameters: severity of inflammation, inflammation extent, crypt damage, and percent involvement. Each parameter is graded from 0 to 3 according to the severity of colonic changes. The sum of these tissue damage scores was then multiplied by a factor corresponding to the fraction of the tissue affected: 1, <10%; 2, 10%−25%; 3, 25%−50%; and 4, >50%. This semiquantitative composite histological pathology scoring system results in tissue damage scores ranging from 0 to 36. A higher score means that the inflammation is more severe in the colon.

### Immunohistochemistry

Mice colon tissue was fixed in 4% paraformaldehyde and processed into paraffin-embedded sections (4 µm thickness). Paraffin sections were subjected to heat-induced antigen retrieval using 10 mM sodium citrate buffer (pH 6.0). Sections were blocked with 5% bovine serum albumin (BSA) and then incubated overnight at 4°C with an anti-TNF-α primary antibody (Santa, sc-12744, 1:200 dilution). After washing, sections were incubated with a biotinylated secondary antibody (1:500 dilution) followed by the streptavidin-HRP conjugate. Visualization was achieved using a DAB substrate, and sections were counterstained with hematoxylin. Stained sections were examined under a light microscope, and images were captured at appropriate magnifications.

TNF-α expression levels were quantified using ImageJ analysis software.

Data analysis was performed using statistical software, and results were presented graphically.

### Statistical analysis

Statistical analysis was done using GraphPad Prism 5 software (https://www.graphpad.com/). Data are presented as mean ± standard error of the mean (SEM). The differences between the control and experimental groups were analyzed using one-way or two-way analysis of variance (ANOVA) and *t*-tests. A *P*-value of ≤0.05 was considered statistically significant.

## SUPPLEMENTAL MATERIAL

Figure S1 mbio.02524-24-s0001.tiff The SENP1-SIRT3 axis suppresses inflammation by inhibiting glycolysis, as related to Fig. 3.

Figure S2 mbio.02524-24-s0002.tiff The expression of TNF-α in different mouse models.

Figure S3 mbio.02524-24-s0003.pdf Original Western blot data from this study.

Legends mbio.02524-24-s0004.docx Supplemental figure legends.

Table S1 mbio.02524-24-s0005.docx Summary of sequencing data.

Table S2 mbio.02524-24-s0006.docx Summary of antibody data.

ASM does not own the copyrights to Supplemental Material that may be linked to, or accessed through, an article. The authors have granted ASM a non-exclusive, world-wide license to publish the Supplemental Material files. Please contact the corresponding author directly for reuse.
