# Supplementary figures and images for: SENP1-SIRT3 axis mediates glycolytic reprogramming to suppress inflammation during Listeria monocytogenes infection
Source: mBio. 2025 Mar 12;16(4):e02524-24. doi: 10.1128/mbio.02524-24 (PMC11980586; doi:10.1128/mbio.02524-24)

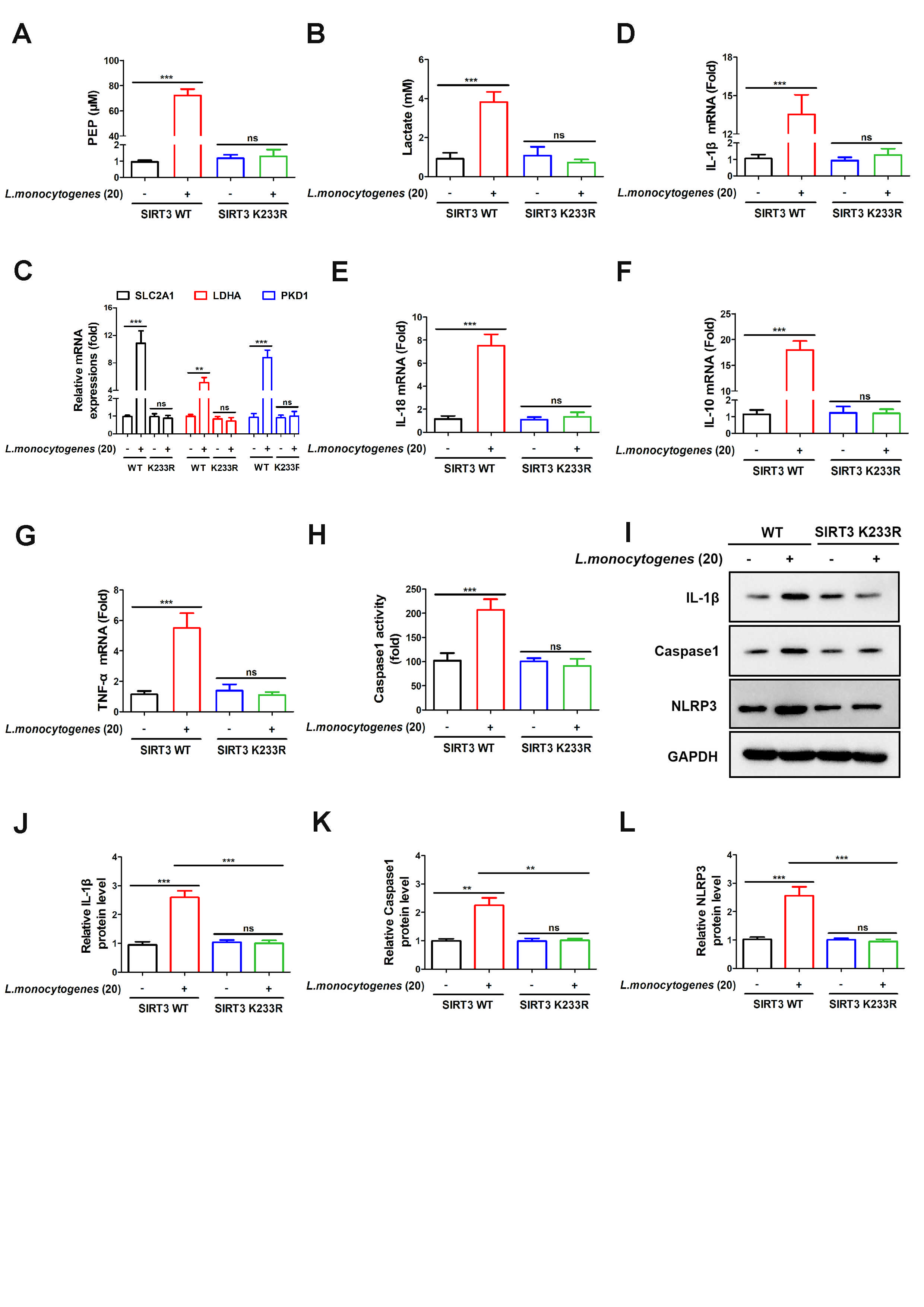

Supplement: Figure S1 — The SENP1-SIRT3 axis suppresses inflammation by inhibiting glycolysis, as related to Fig. 3. [file mbio.02524-24-s0001.tiff]

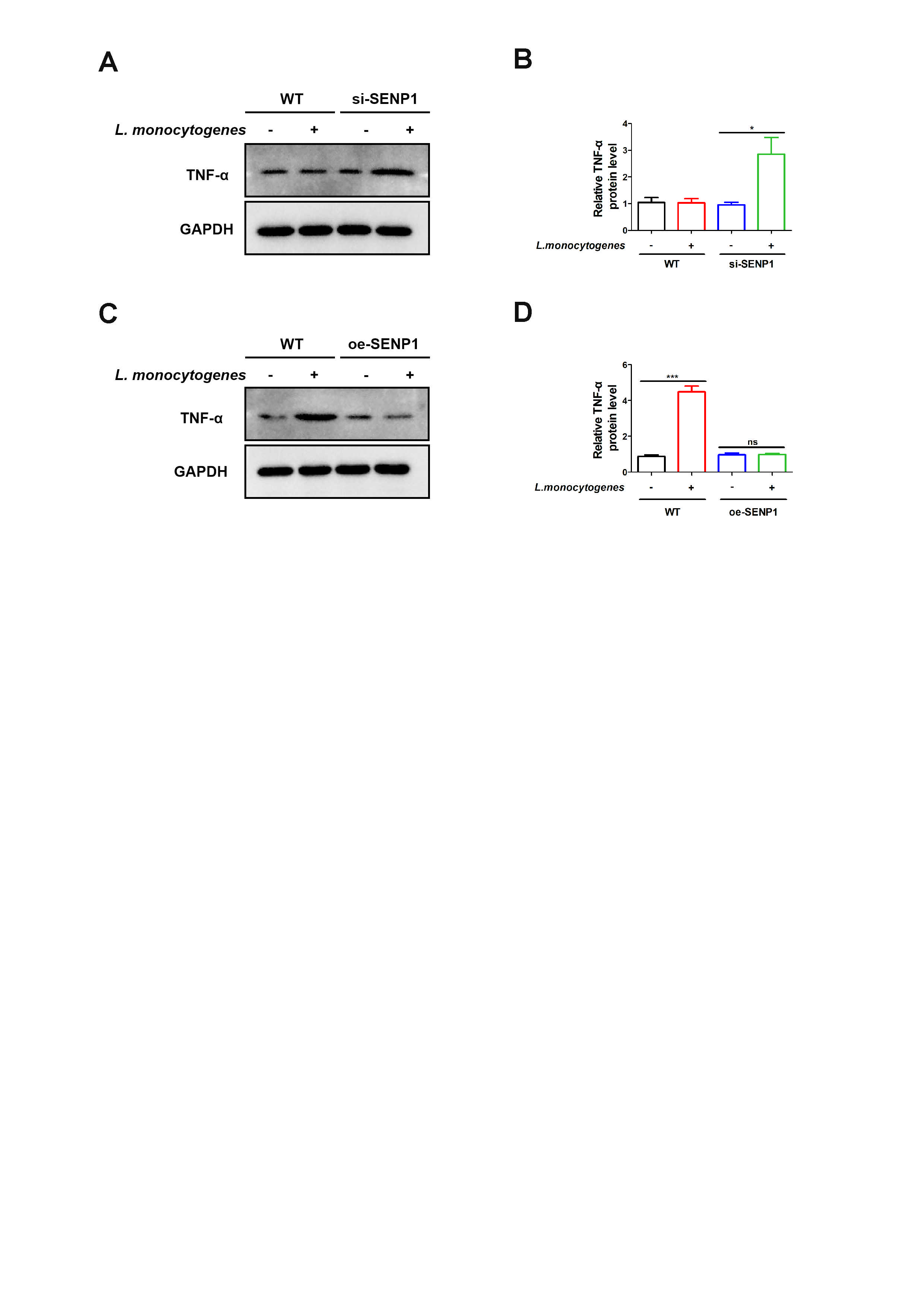

Supplement: Figure S2 — The expression of TNF-α in different mouse models. [file mbio.02524-24-s0002.tiff]
