# Supplementary material for: SENP1-SIRT3 axis mediates glycolytic reprogramming to suppress inflammation during Listeria monocytogenes infection
Source: mBio. 2025 Mar 12;16(4):e02524-24. doi: 10.1128/mbio.02524-24 (PMC11980586; doi:10.1128/mbio.02524-24)

Fig 1I

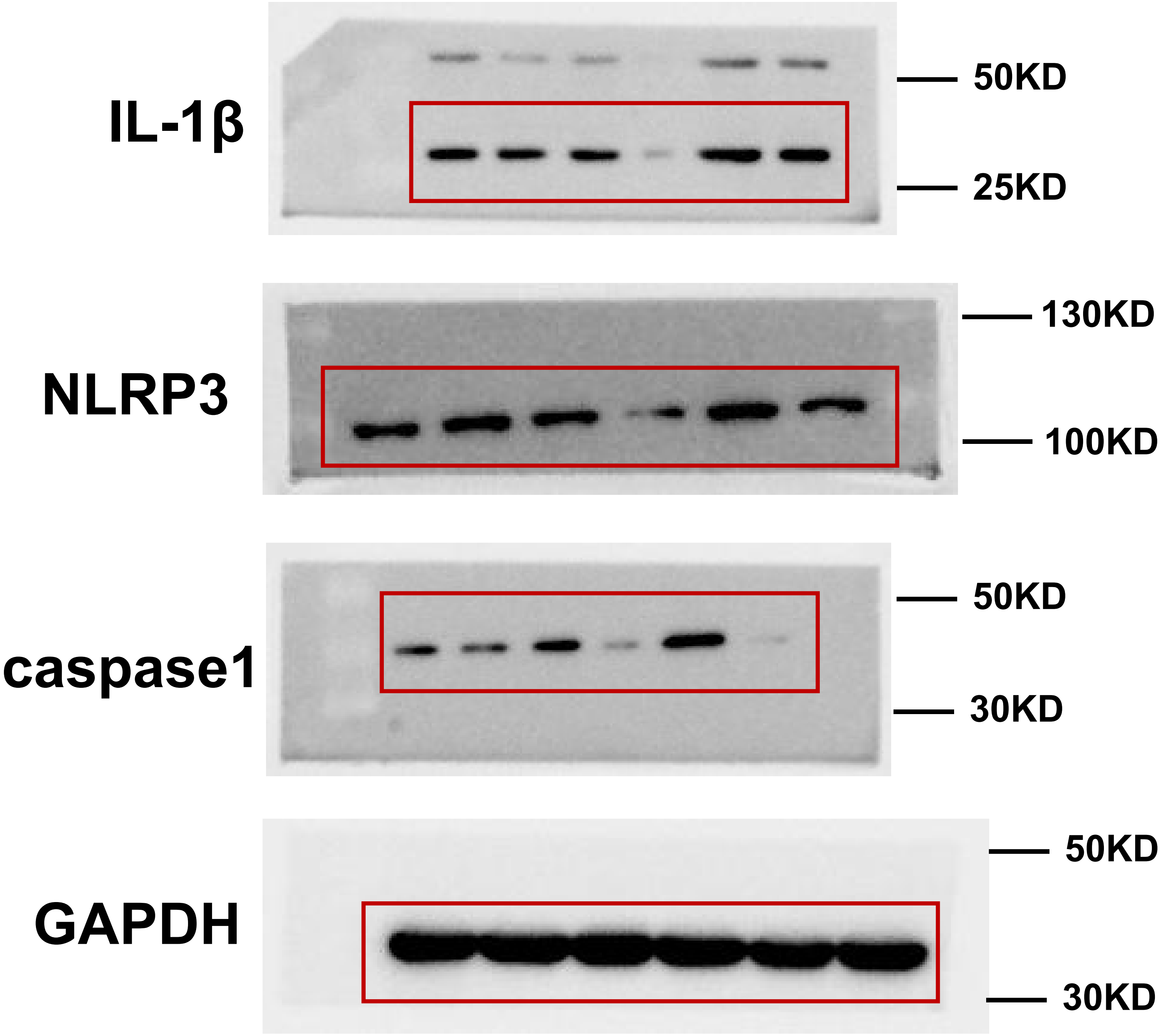
IL-1β
50KD
25KD
NLRP3
130KD
100KD
caspase1
50KD
30KD
GAPDH
50KD
30KD

Fig 2A

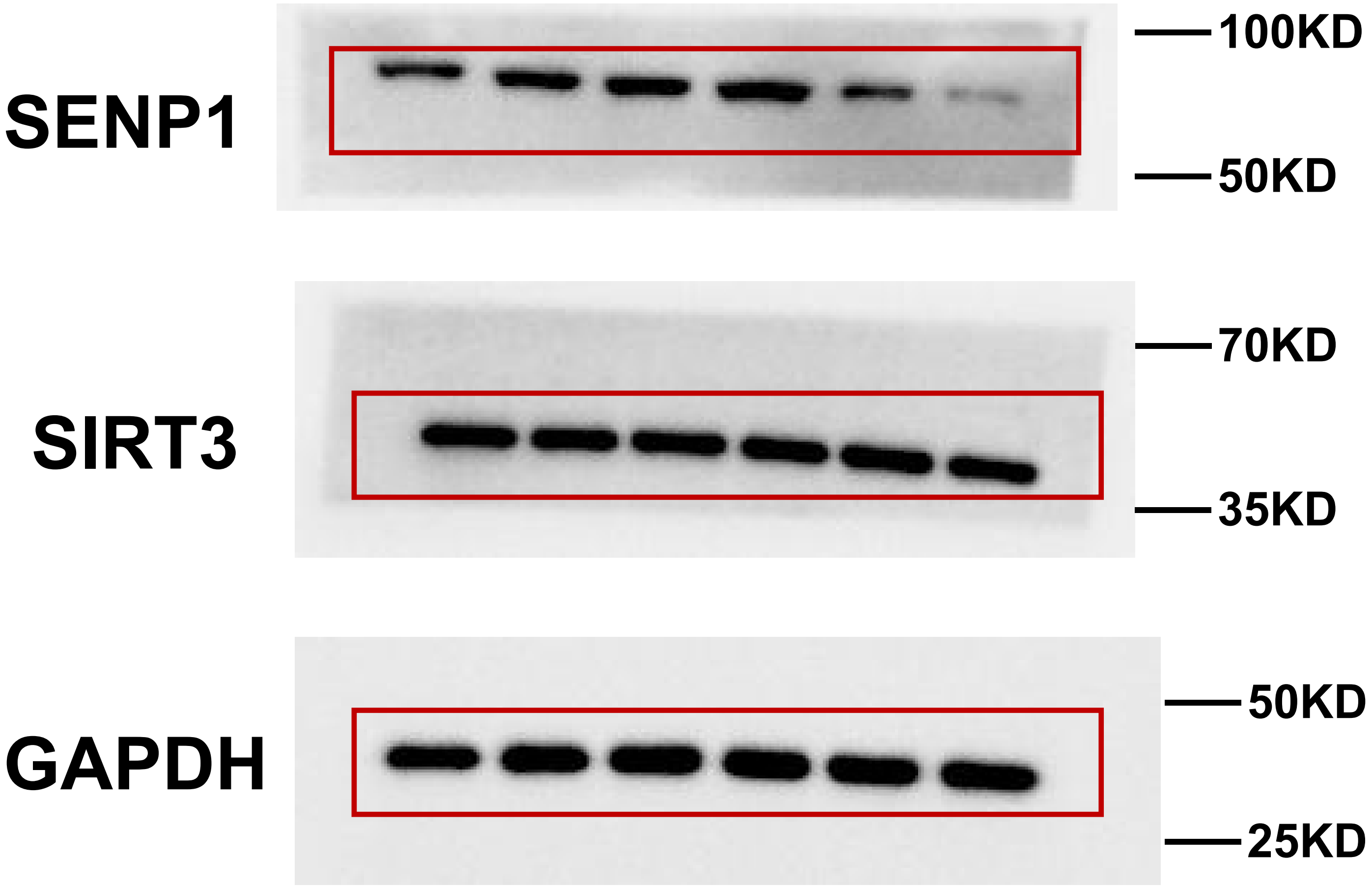

Fig 2C

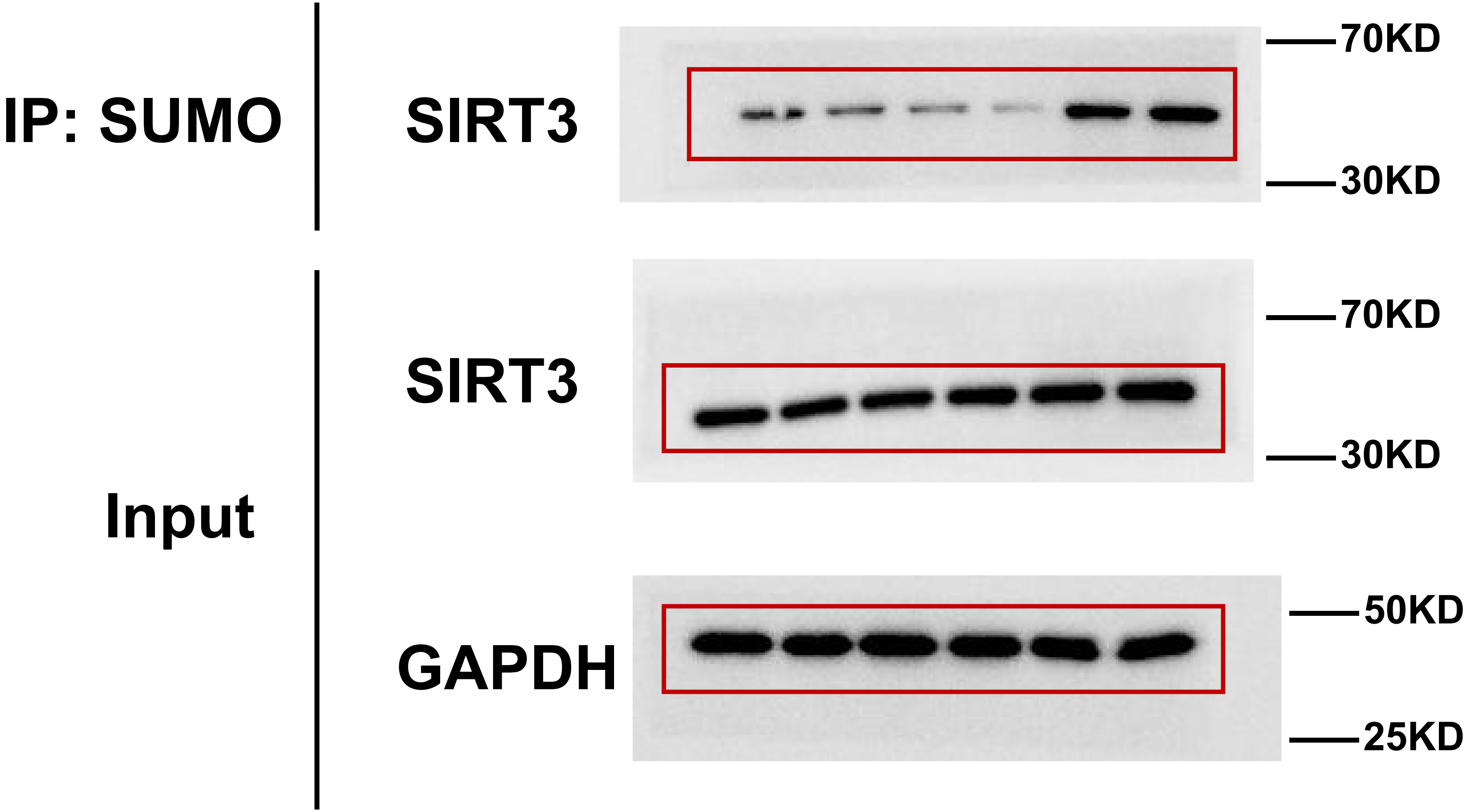
IP: SUMO
SIRT3
70KD
30KD
Input
SIRT3
70KD
30KD
GAPDH
50KD
25KD

Fig 2E

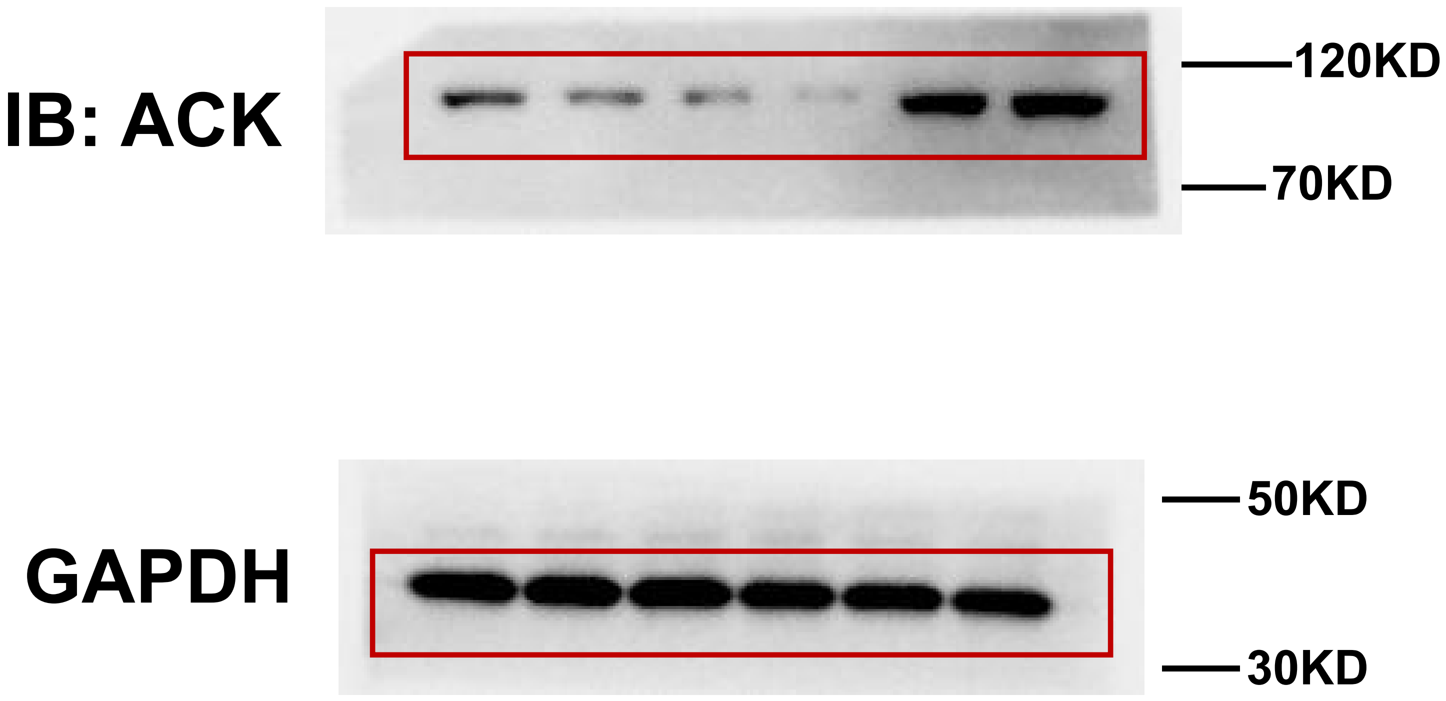

Fig 2G

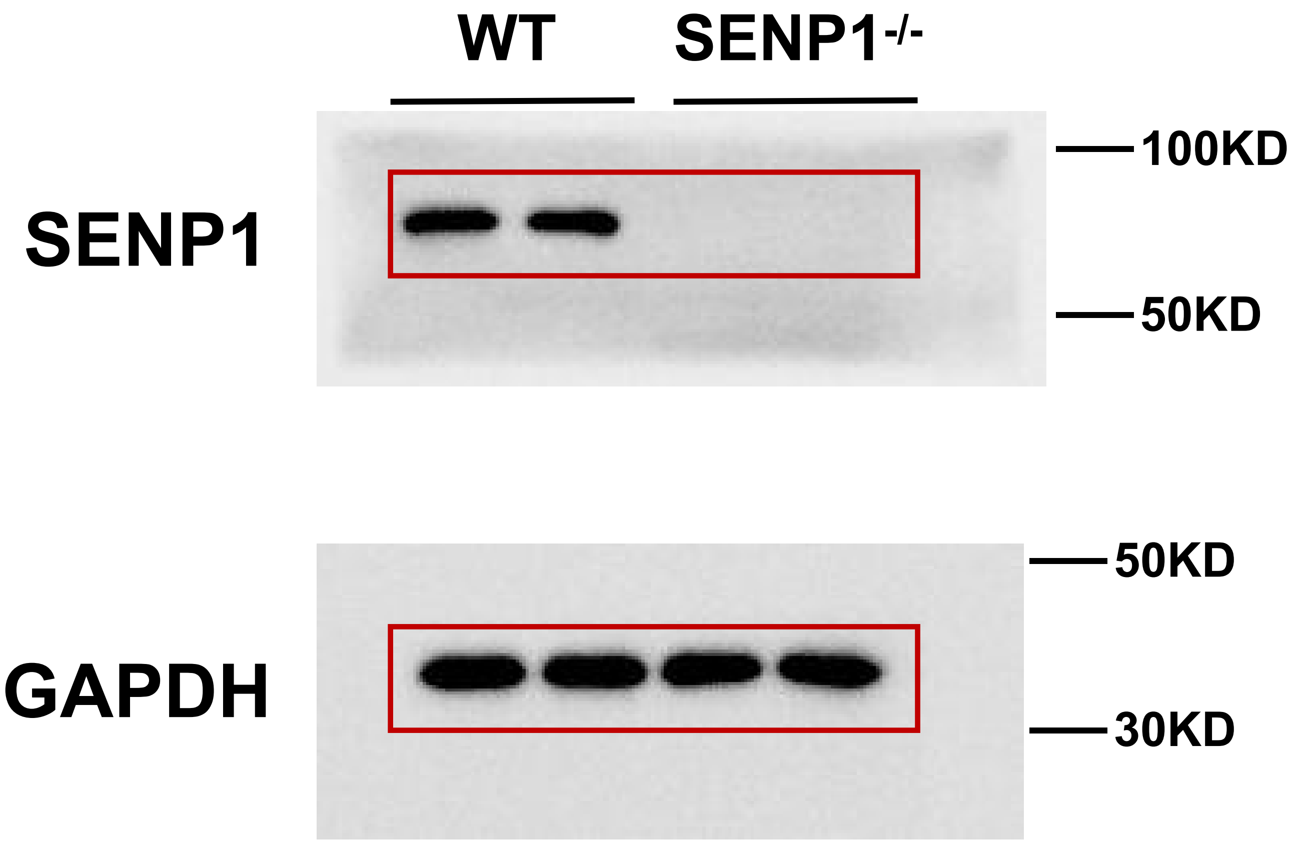

Fig 2H

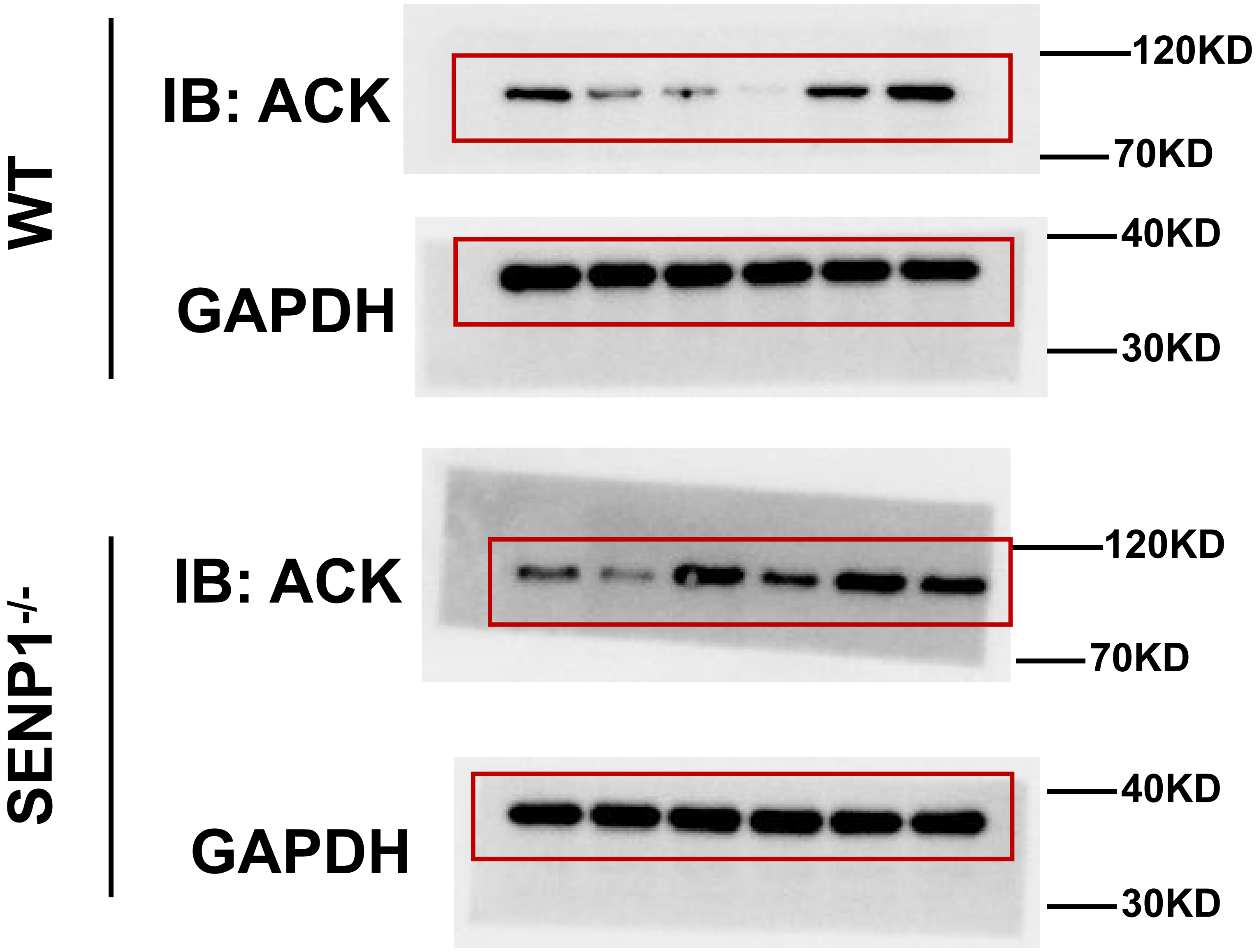

WT
IB: ACK
120KD
70KD
GAPDH
40KD
30KD
SENP1-/-
IB: ACK
120KD
70KD
GAPDH
40KD
30KD

Fig 2J

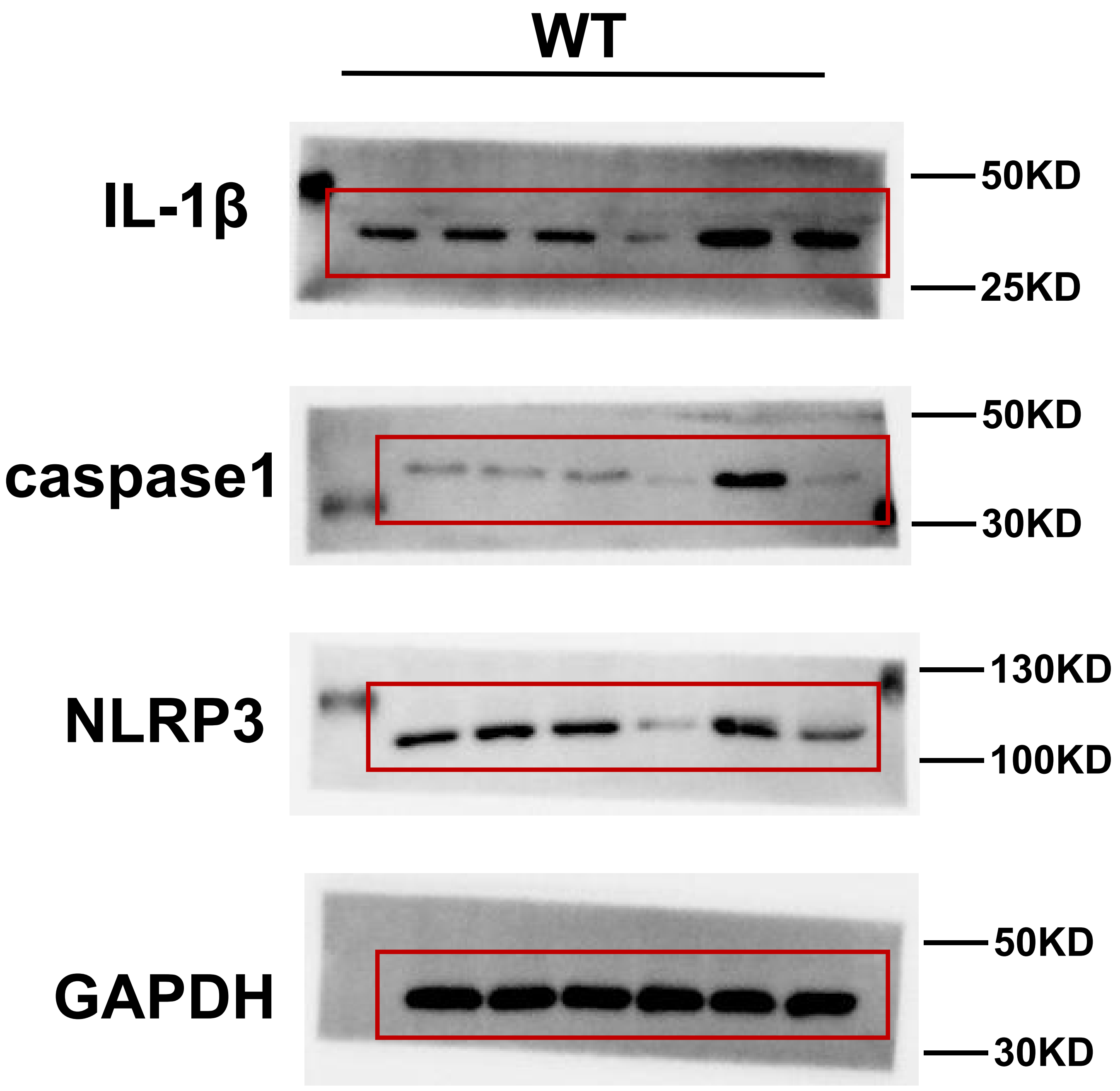
WT
IL-1β
50KD
25KD
caspase1
50KD
30KD
NLRP3
130KD
100KD
GAPDH
50KD
30KD

Fig 2J

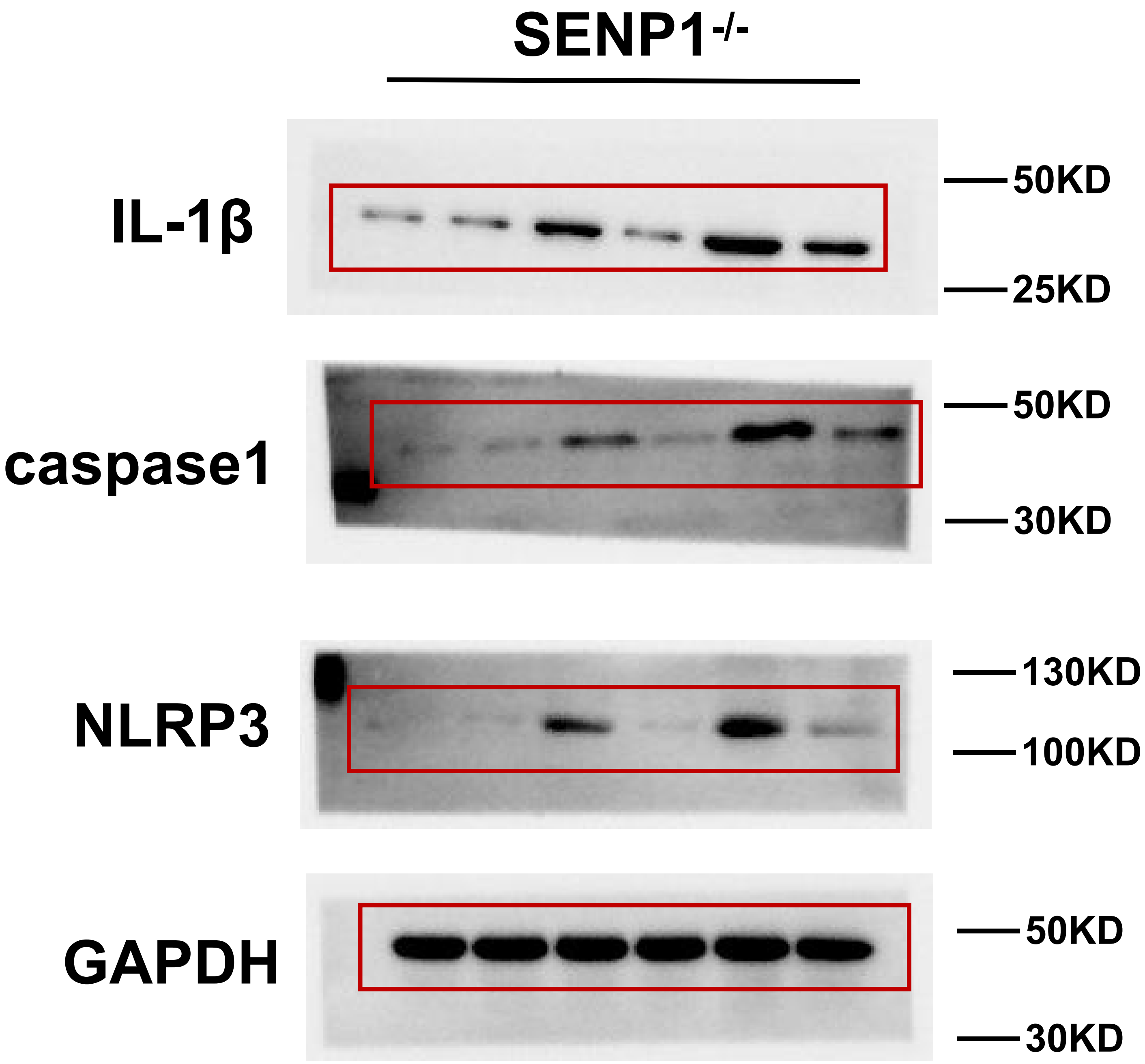
SENP1-/-
IL-1β
caspase1
NLRP3
GAPDH
50KD
25KD
50KD
30KD
130KD
100KD
50KD
30KD

Fig 3I

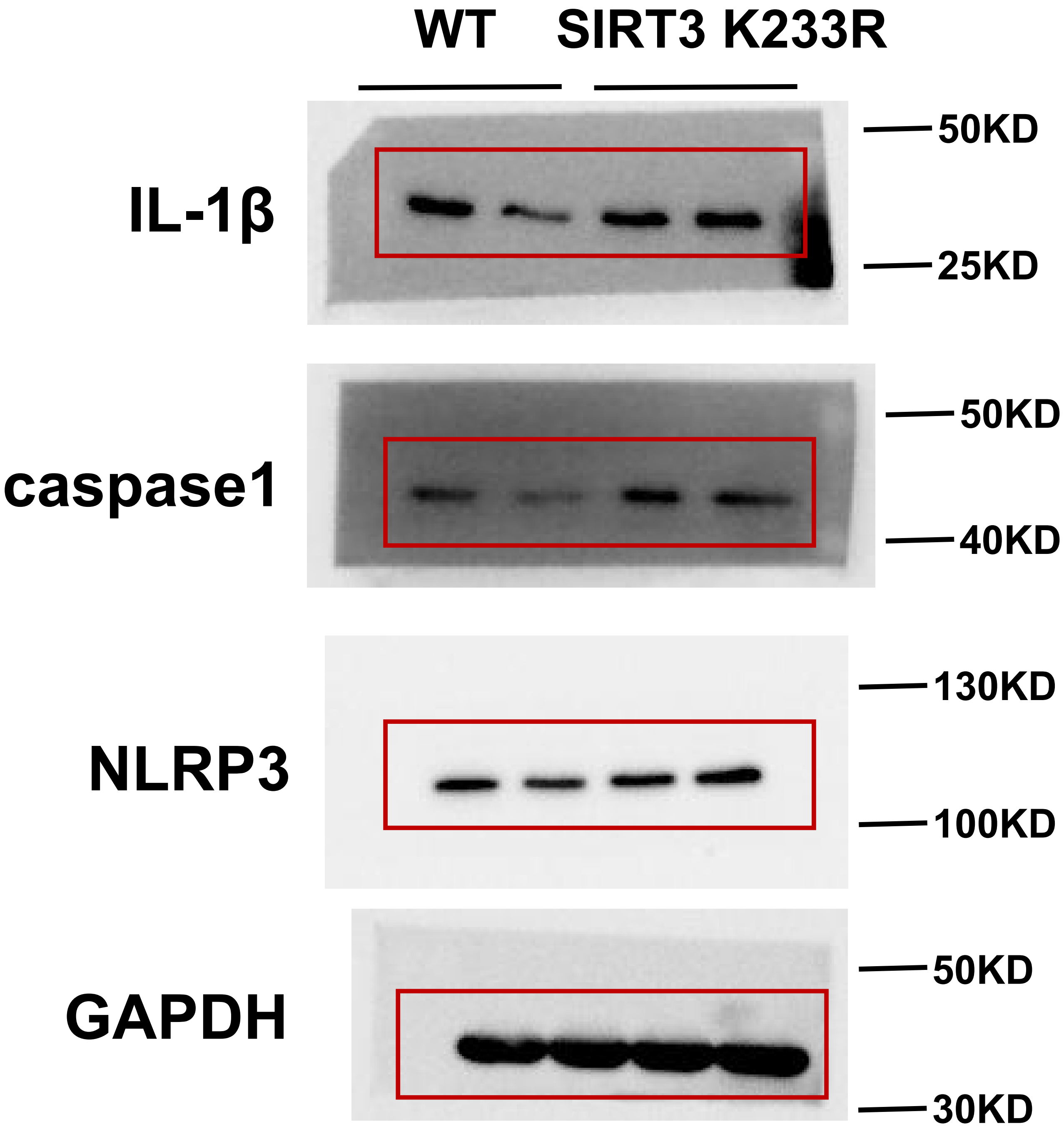

WT
SIRT3 K233R
IL-1β
50KD
25KD
caspase1
50KD
40KD
NLRP3
130KD
100KD
GAPDH
50KD
30KD

Fig 3-Sup1-3I

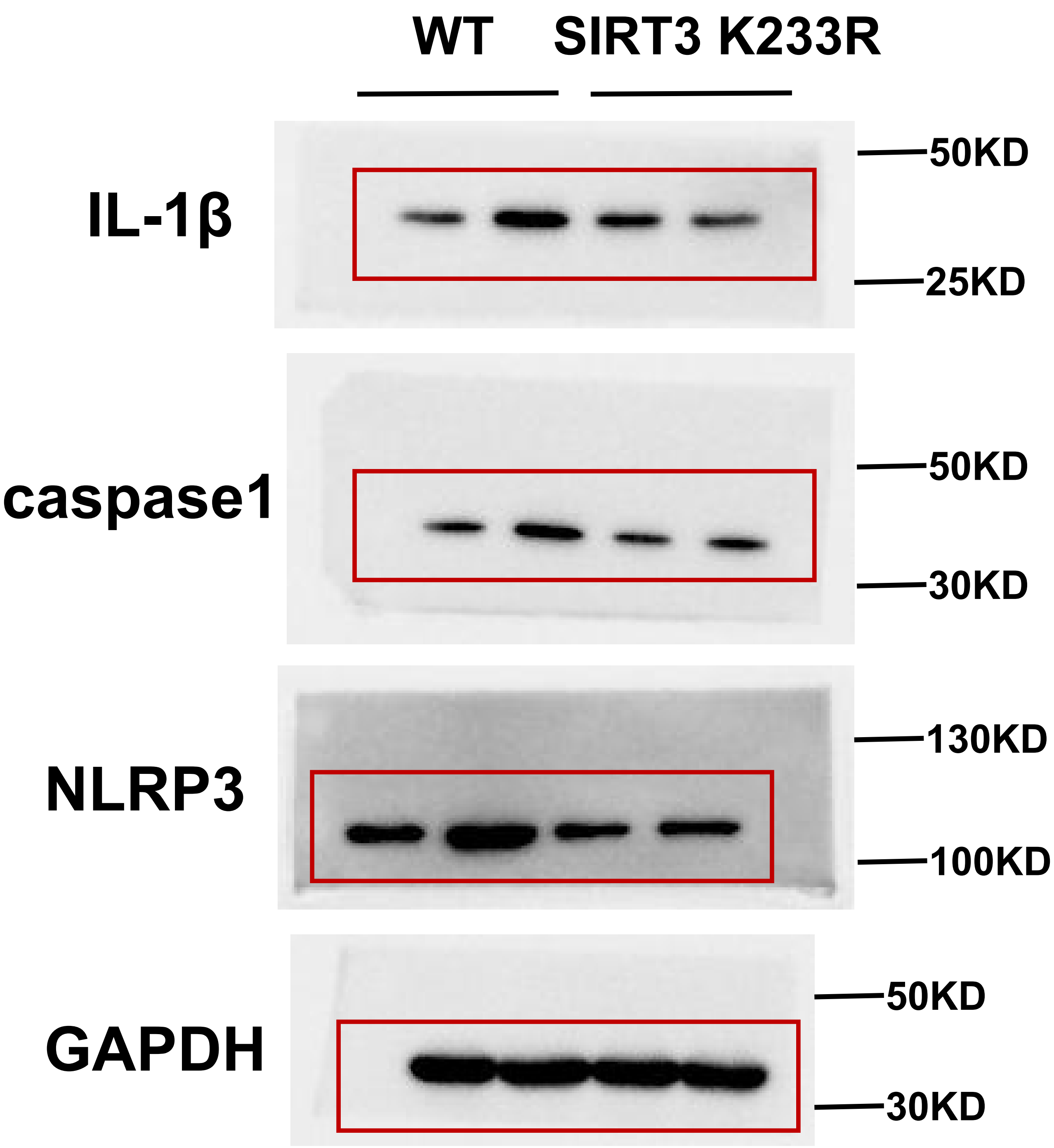
WT
SIRT3 K233R
IL-1β
caspase1
NLRP3
GAPDH
50KD
25KD
50KD
30KD
130KD
100KD
50KD
30KD

Fig 4A

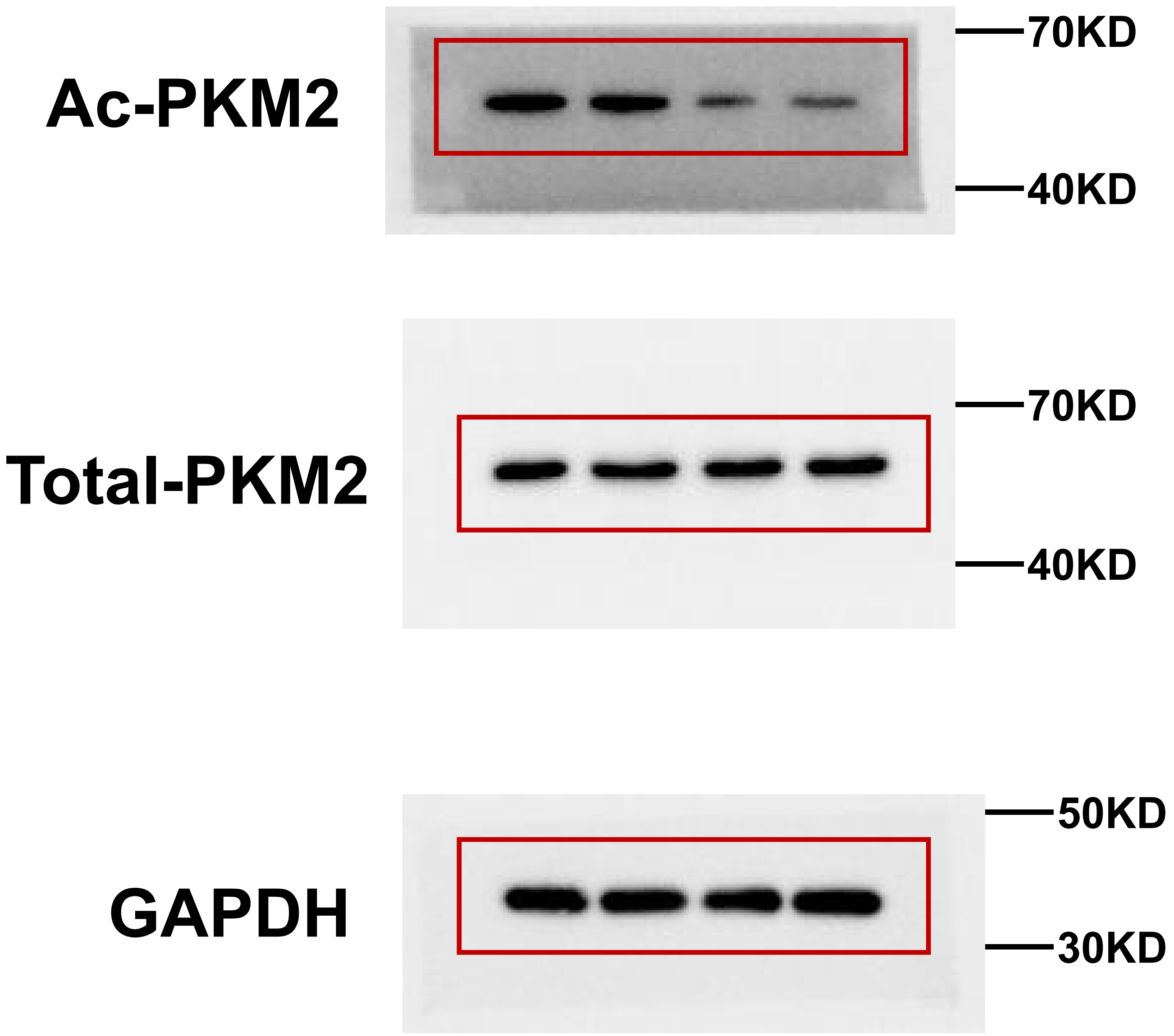

Fig 4D

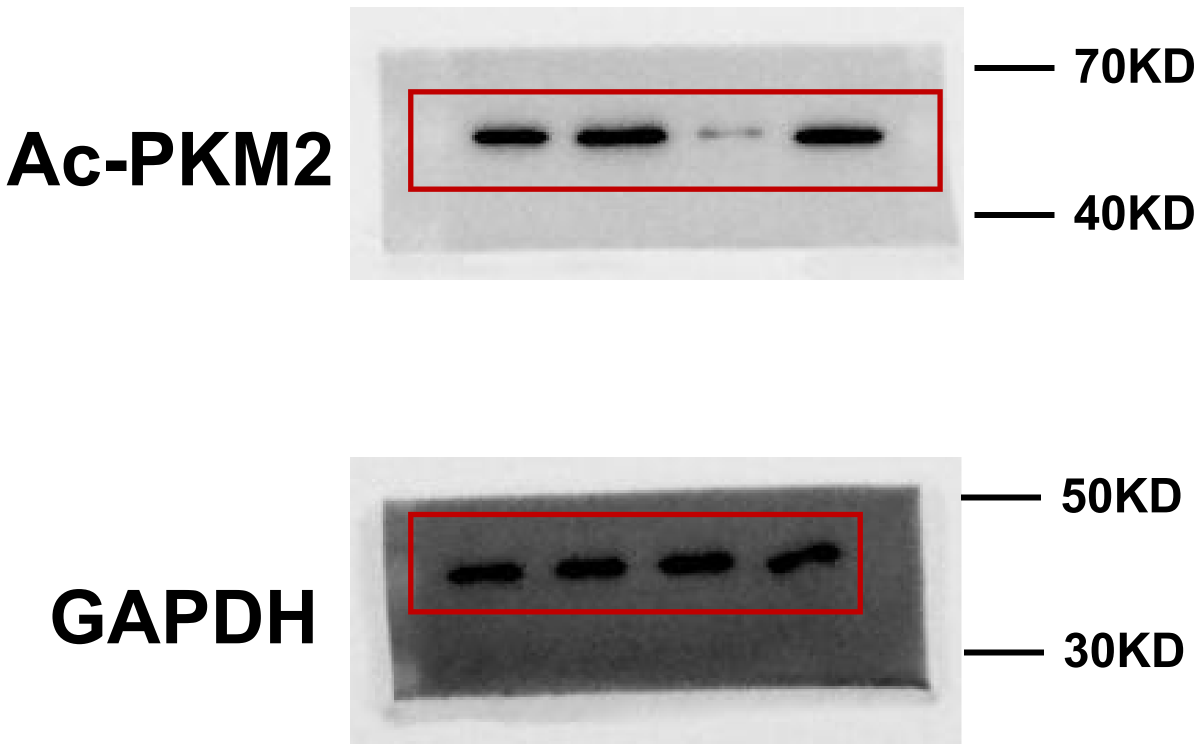
Ac-PKM2
70KD
40KD
GAPDH
50KD
30KD

Fig4F

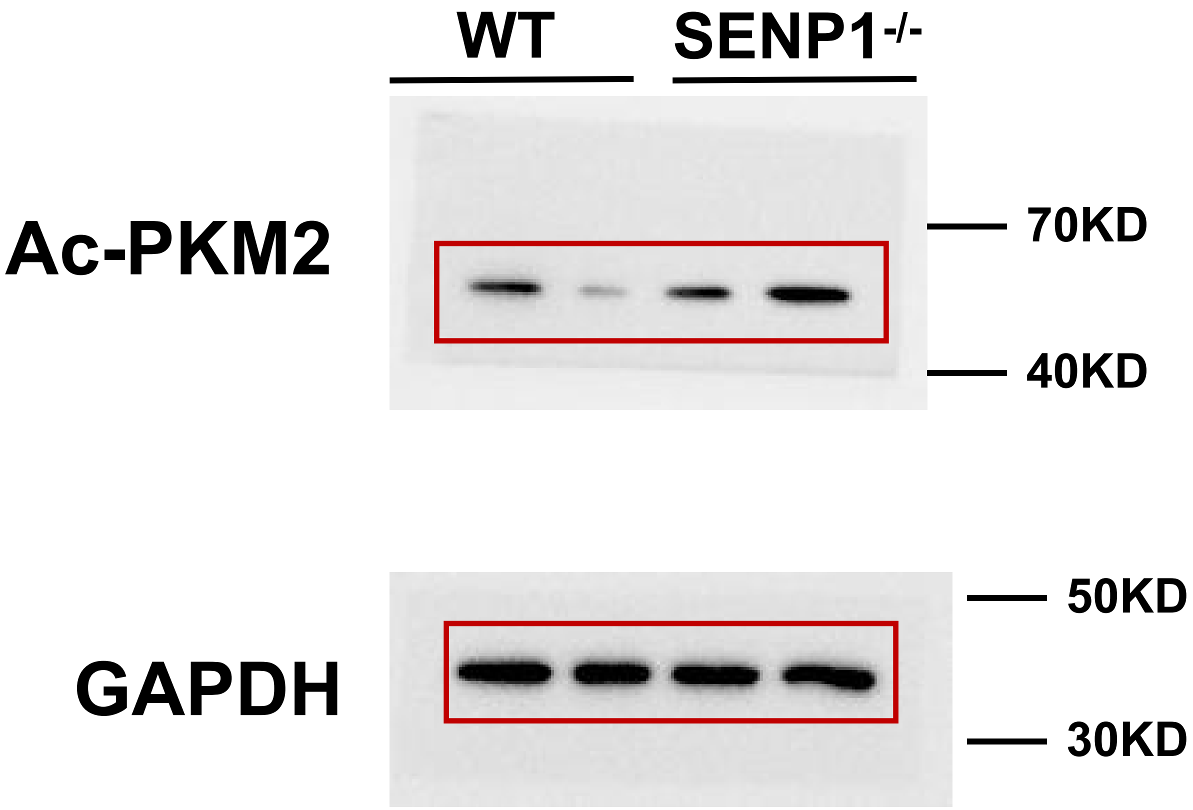
WT
SENP1-/-
Ac-PKM2
70KD
40KD
GAPDH
50KD
30KD

Fig 5F

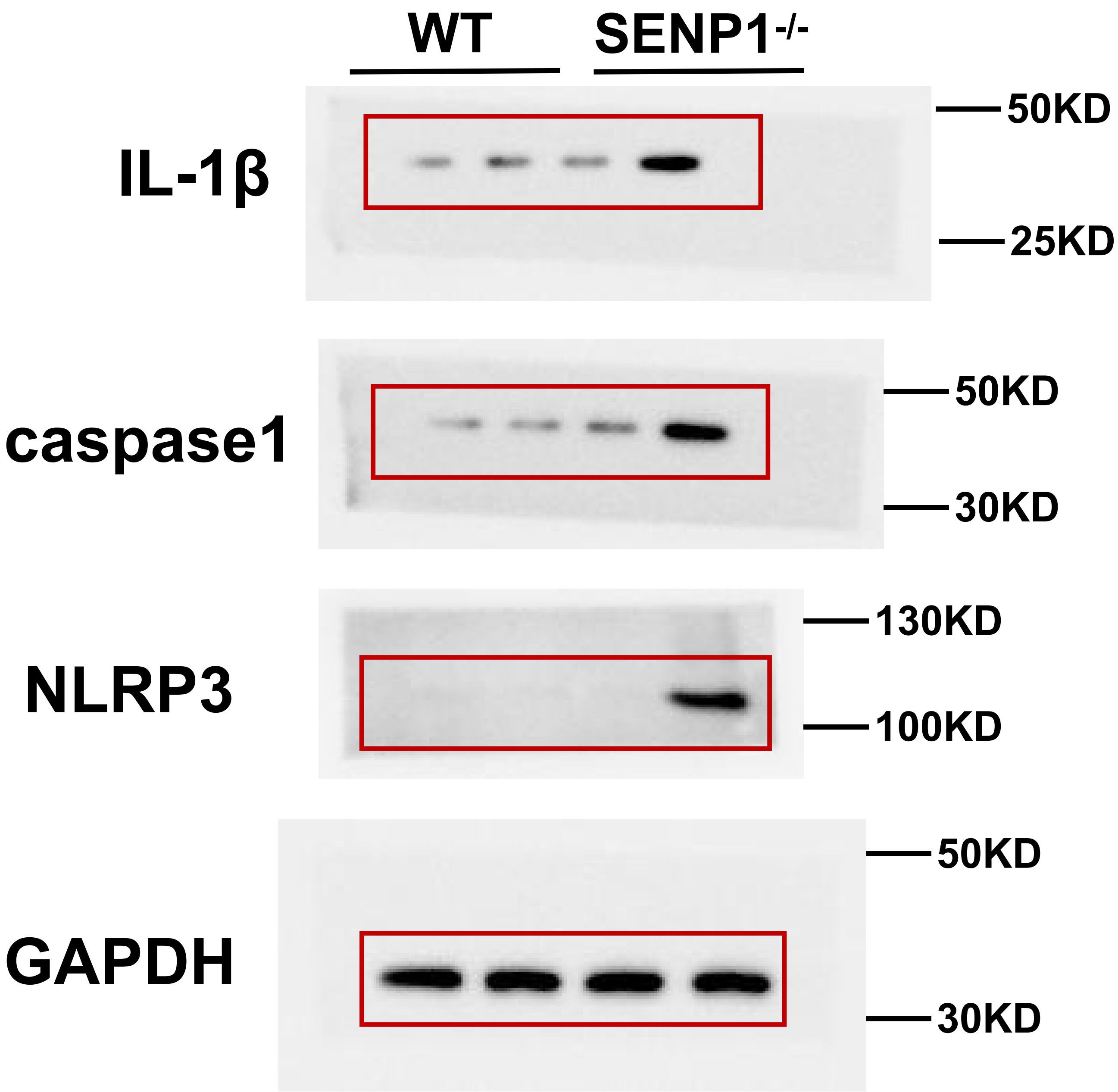

WT
SENP1-/-
IL-1β
caspase1
NLRP3
GAPDH
50KD
25KD
50KD
30KD
130KD
100KD
50KD
30KD

Fig 5I

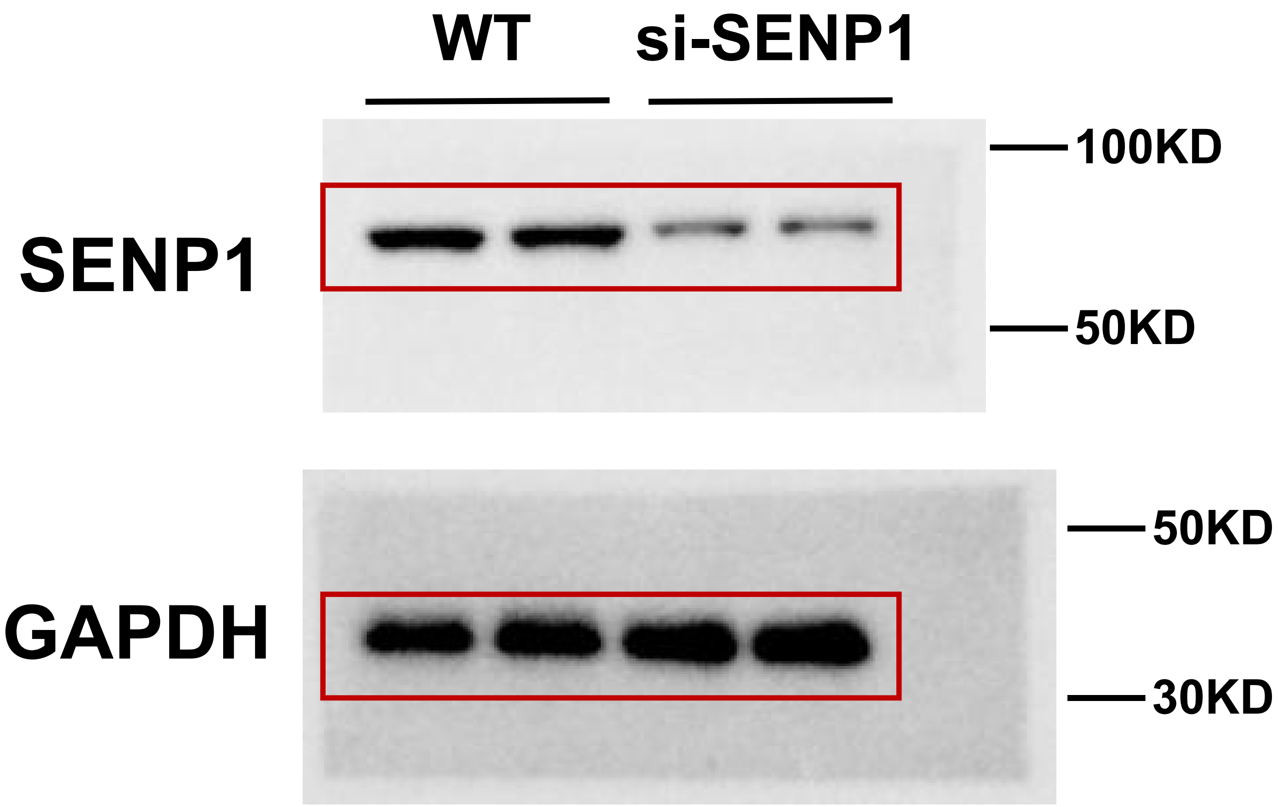

Fig 5K

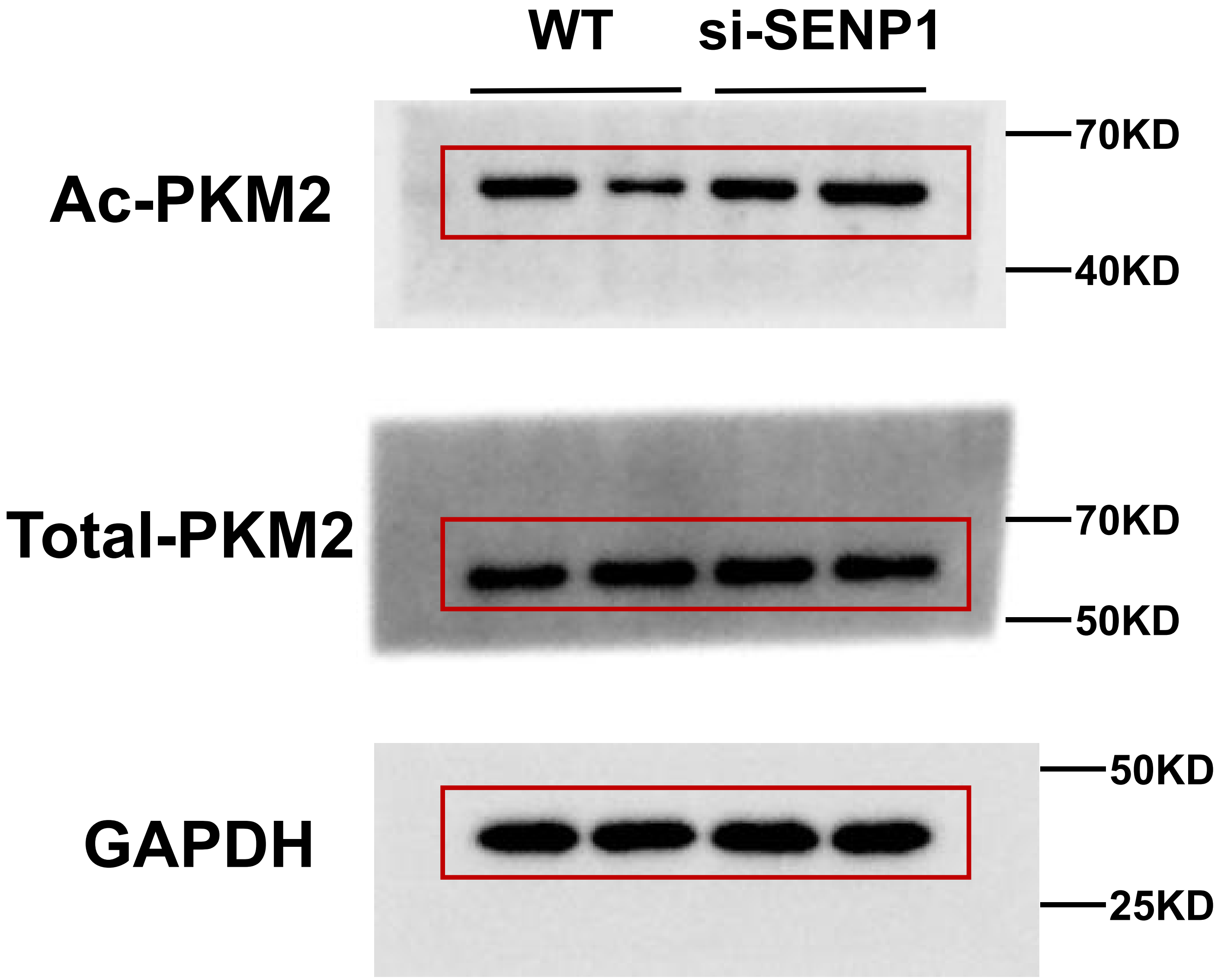

WT
si-SENP1
Ac-PKM2
70KD
40KD
Total-PKM2
70KD
50KD
GAPDH
50KD
25KD

Fig 6F

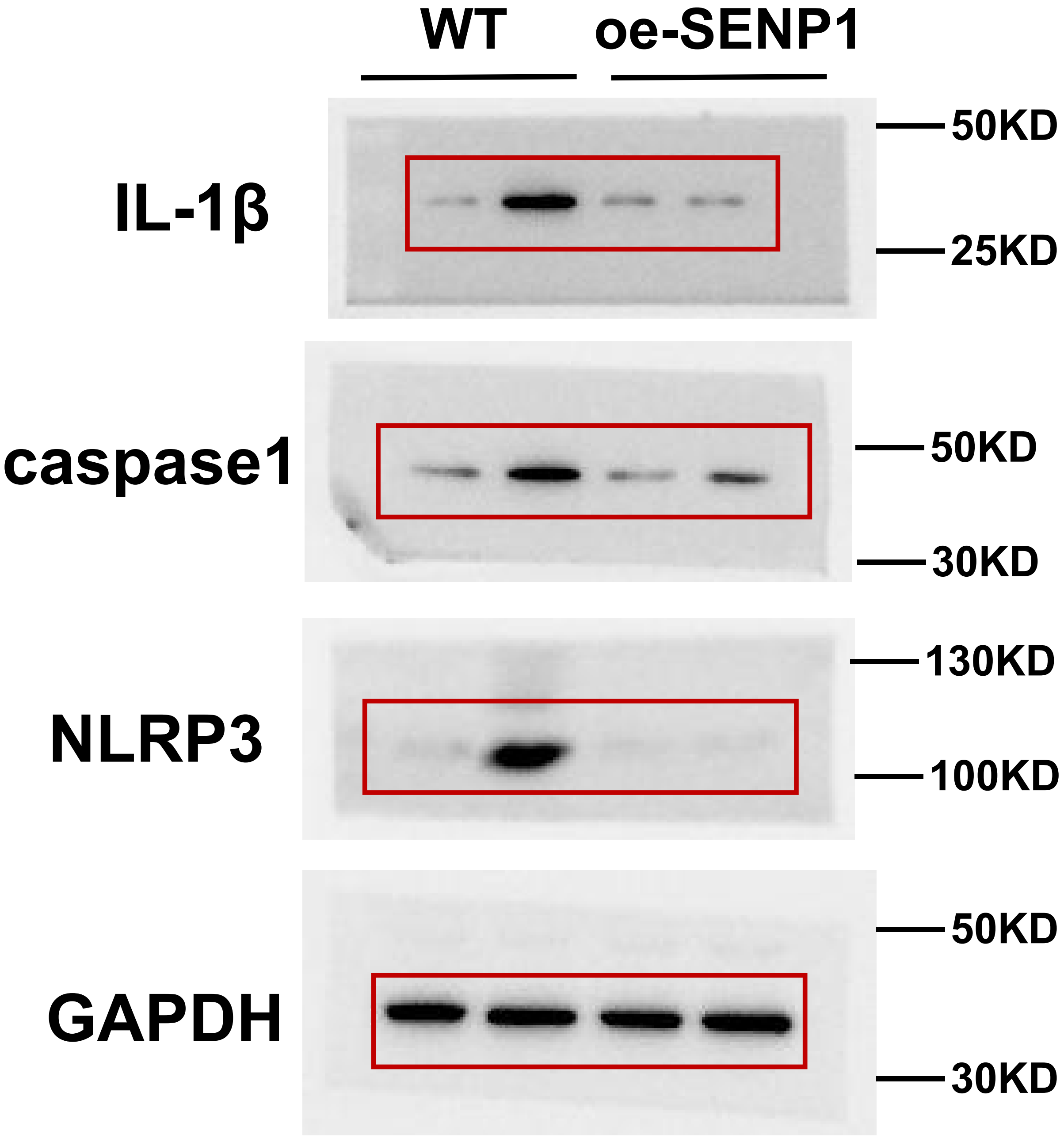

WT
oe-SENP1
IL-1β
caspase1
NLRP3
GAPDH
50KD
25KD
50KD
30KD
130KD
100KD
50KD
30KD

Fig 6I

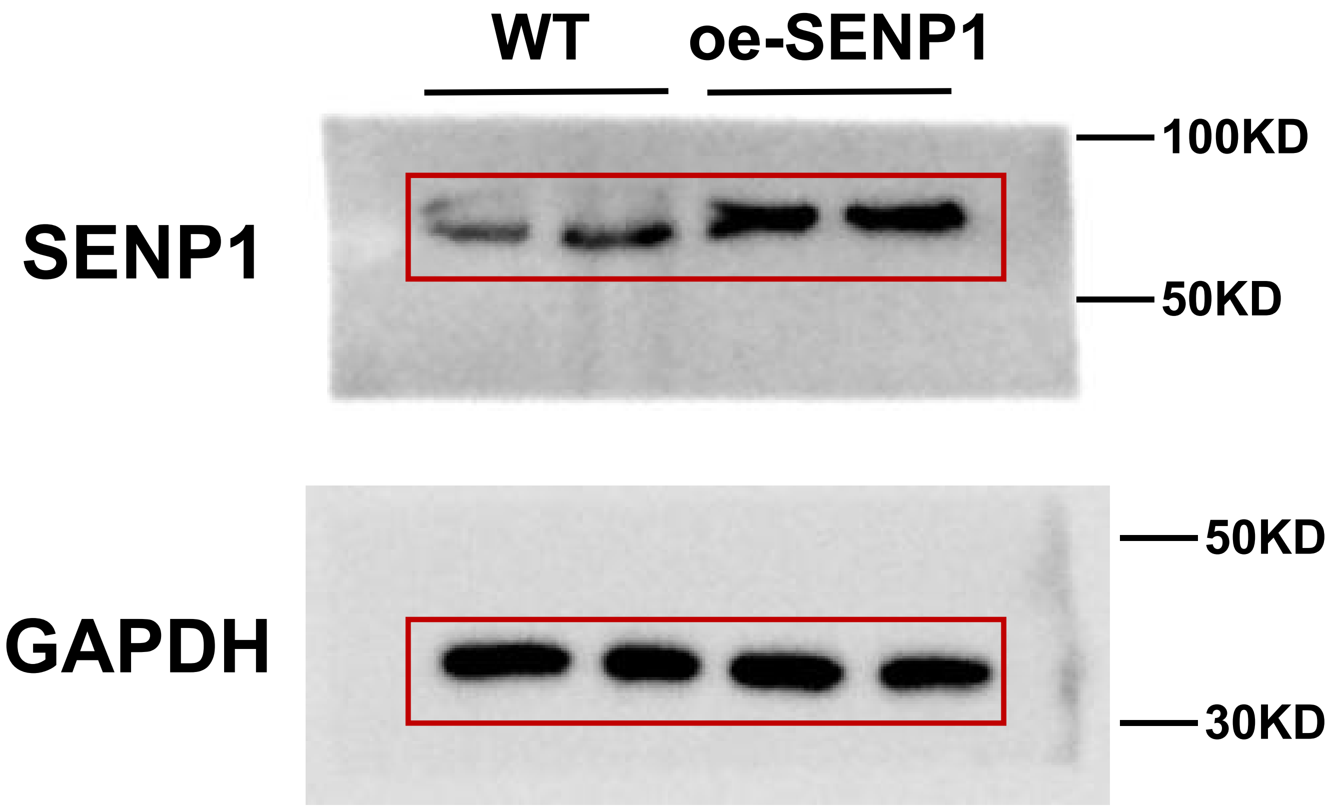

Fig 6K

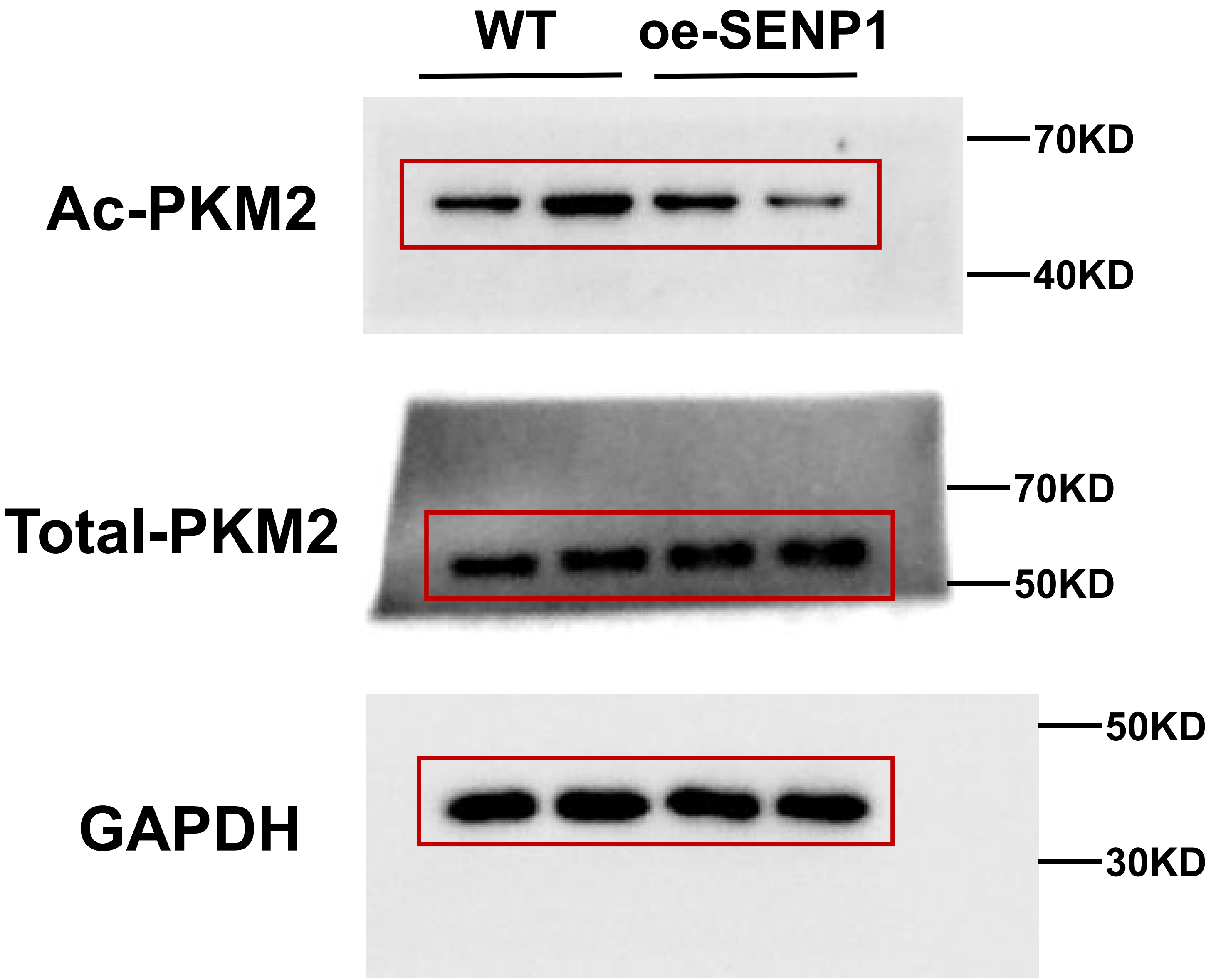

WT
oe-SENP1
Ac-PKM2
70KD
40KD
Total-PKM2
70KD
50KD
GAPDH
50KD
30KD

Fig 5, 6-Sup2- A， B

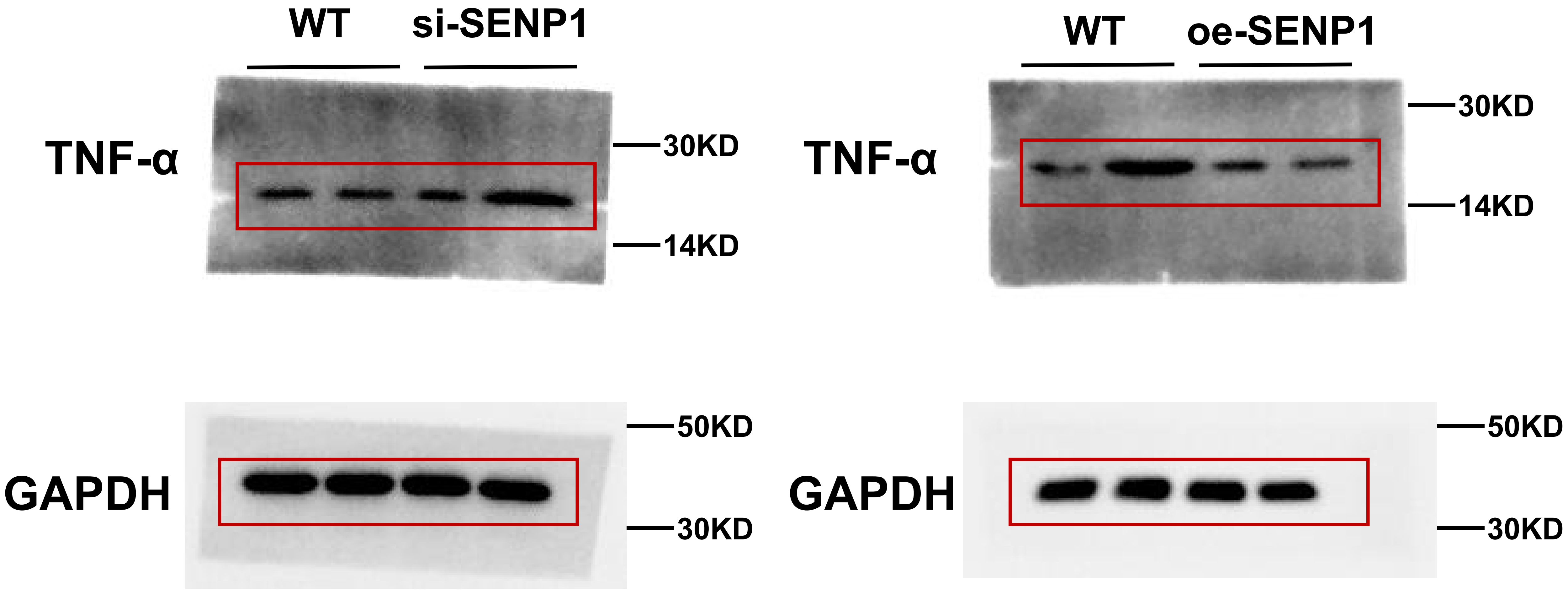

Supplement: Figure S3 — Original Western blot data from this study. [file mbio.02524-24-s0003.pdf]
