# Supplementary material for: SENP1-SIRT3 axis mediates glycolytic reprogramming to suppress inflammation during Listeria monocytogenes infection
Source: mBio. 2025 Mar 12;16(4):e02524-24. doi: 10.1128/mbio.02524-24 (PMC11980586; doi:10.1128/mbio.02524-24)
Supplement: Legends — Supplemental figure legends. [file mbio.02524-24-s0004.docx]

**Supplemental legends**

**Figure S1. The SENP1-SIRT3 axis suppresses inflammation by inhibiting glycolysis, as related to Figure 3**

(A) Caco-2 cells (SIRT3 WT, SIRT3 K233R) were treated with or without *L. monocytogenes* (MOI = 20) for 12 h. Glycolytic activity was measured using the Glycolysis Assay kit. (B) Caco-2 cells (SIRT3 WT, SIRT3-K233R) were infected with or without *L. monocytogenes* (MOI = 20) for 12 h, and then the lactate levels were measured by the lactate detection kit. (C) Caco-2 cells (SIRT3 WT, SIRT3-K233R) were infected with or without *L. monocytogenes* (MOI = 20) for 12 h. The expression levels of SLC2A1, LDHA, and PKD1 genes were determined by Real-time quantitative PCR in each cell group. (D-G) Wild-type (WT) and SIRT3-K233R Caco-2 cells were infected with or without *L. monocytogenes* (MOI = 20) for 12 h, and then the expression levels of IL-1β, IL-18, IL-10, and TNF-α were assessed by Real-time quantitative PCR in different cell groups. (H) Wild-type (WT) and SIRT3-K233R Caco-2 cells were infected with or without *L. monocytogenes* (MOI = 20) for 12 h. The Caspase 1 activity was assessed. (I) Wild-type (WT) and SIRT3-K233R Caco-2 cells were infected with or without *L. monocytogenes* (MOI = 20) for 12 h, and then the IL-1β, Caspase 1, and NLRP3 expressions were analyzed by Western blotting. (J-L) Statistical results for (I).

Data shown in A, B, D-H, J-L were analyzed by one-way ANOVA. Data shown in C was analyzed by two-way ANOVA. The blots represented three independent experiments. All data are presented as the mean ± SEM of n = 6. *** p < 0.001, ns,  **Figure S2. The expression of TNF-α in different mouse models**

(A) Mice (WT, si-SENP1) were treated with *L. monocytogenes* (3 × 10^4^ CFU, 200 μL, i.g.). Colonic tissue proteins were extracted, and then the expression levels of TNF-α were assessed by immunoblotting analysis. (B) Statistical results for (A). (C) Mice (WT, oe-SENP1) were treated with *L. monocytogenes* (3 × 10^9^ CFU, 200 μL, i.g.). Colonic tissue proteins were extracted, and then the expression levels of TNF-α were assessed by immunoblotting analysis. (D) Statistical results for (C).

**Figure S3. Original Western blot data from this study.**

The raw Western blot images show the expression levels of target proteins under experimental conditions. Protein samples were separated by SDS-PAGE, transferred to PVDF membranes, and probed with specific primary antibodies followed by HRP-conjugated secondary antibodies. The blots were developed using chemiluminescence. Molecular weight markers (in kDa) are indicated on the right. The images are presented without modification.
