# Supplementary material for: SENP1-SIRT3 axis mediates glycolytic reprogramming to suppress inflammation during Listeria monocytogenes infection
Source: mBio. 2025 Mar 12;16(4):e02524-24. doi: 10.1128/mbio.02524-24 (PMC11980586; doi:10.1128/mbio.02524-24)
Supplement: Table S1 — Summary of sequencing data. [file mbio.02524-24-s0005.docx]

Supplementary Table 1

| **primer names** | **Primer sequences(5'-3')** |
| --- | --- |
| IL-10-F | TTCTTTCAAACAAAGGACCAGC |
| IL-10-R | GCAACCCAAGTAACCCTTAAAG |
| IL-12-F | TTTCTAGATGCTGGCCAATACA |
| IL-12-R | ATCTCGGTGGACCAAATTCC |
| IL-6-F | ATCCAGTTGCCTTCTTGGGACTGA |
| IL-6-R | TAAGCCTCCGACTTGTGAAGTGGT |
| IL-1β-F | TTCAGGCAGGCAGTATCACTC |
| IL-1β-R | GAAGGTCCACGGGAAAGACAC |
| IL-18-F | GACTCTTGCGTCAACTTCAAGG |
| IL-18-R | CAGGCTGTCTTTTGTCAACGA |
| TNF-α-F | GAGAAGTTCCCAAATGGC |
| TNF-α-R | ACTTGGTGGTTTGCTAC |
| ACTIN-F | CTACCTCATGAAGATCCTGACC |
| ACTIN-R | CACAGCTTCTCTTTGATGTCAC |
| Promoter-F | ATATGGATCCGAGGGAACTGGCCAA |
| Promoter-R | TATATCTAGATCACCCACTGTTGCCTA |
| SENP1-F | ATATTCTAGATTATGAAAACCCCCTT |
| SENP1-R | TATAGCGGCCGCTCACAAGAGCTT |

**Table S1. Summary of sequencing data.**

The table shows the primer sequences utilized for qPCR.
