# Supplementary material for: SENP1-SIRT3 axis mediates glycolytic reprogramming to suppress inflammation during Listeria monocytogenes infection
Source: mBio. 2025 Mar 12;16(4):e02524-24. doi: 10.1128/mbio.02524-24 (PMC11980586; doi:10.1128/mbio.02524-24)
Supplement: Table S2 — Summary of antibody data. [file mbio.02524-24-s0006.docx]

| **Antibody** | **Dilution** | **Source** | **Catalog number** |
| --- | --- | --- | --- |
| IL-1β | 1:1000 | NOVUS | [AF-401-NA](https://www.novusbio.com/products/il-1-beta-il-1f2-antibody_af-401-na) |
| Caspase-1 | 1:1000 | NOVUS | [NB100-56565](https://www.novusbio.com/products/caspase-1-antibody-14f468_nb100-56565) |
| NLRP3 | 1:1000 | NOVUS | [NBP2-67639](https://www.novusbio.com/products/nlrp3-nalp3-antibody-sc06-23_nbp2-67639) |
| SENP1 | 1:800 | Abcam | ab225887 |
| SIRT3 | 1:1000 | Abcam | ab189860 |
| PKM2 | 1:5000 | Thermo Fisher Scientific | PA5-29339 |
| GAPDH | 1:1000 | Thermo Fisher Scientific | PA1-987 |
| TNF-α | 1:200 | Santa | sc-12744 |

Supplementary Table 2

**Table S2. Summary of antibody data.**

The table summarizes the information on all antibodies used in this study.
